# The α-Crystallin Domain Containing Genes: Identification, Phylogeny and Expression Profiling in Abiotic Stress, Phytohormone Response and Development in Tomato (*Solanum lycopersicum*)

**DOI:** 10.3389/fpls.2016.00426

**Published:** 2016-03-31

**Authors:** Asosii Paul, Sombir Rao, Saloni Mathur

**Affiliations:** National Institute of Plant Genome ResearchNew Delhi, India

**Keywords:** α-crystallin domain, abiotic stress, gene expression, *Solanum lycopersicum*, sHSPs

## Abstract

The α-crystallin domain (ACD) is an ancient domain conserved among all kingdoms. Plant ACD proteins have roles in abiotic stresses, transcriptional regulation, inhibiting virus movement, and DNA demethylation. An exhaustive *in-silico* analysis using Hidden Markov Model-based conserved motif search of the tomato proteome yielded a total of 50 ACD proteins that belonged to four groups, sub-divided further into 18 classes. One of these groups belongs to the small heat shock protein (sHSP) class of proteins, molecular chaperones implicated in heat tolerance. Both tandem and segmental duplication events appear to have shaped the expansion of this gene family with purifying selection being the primary driving force for evolution. The expression profiling of the Acd genes in two different heat stress regimes suggested that their transcripts are differentially regulated with roles in acclimation and adaptive response during recovery. The co-expression of various genes in response to different abiotic stresses (heat, low temperature, dehydration, salinity, and oxidative stress) and phytohormones (abscisic acid and salicylic acid) suggested possible cross-talk between various members to combat a myriad of stresses. Further, several genes were highly expressed in fruit, root, and flower tissues as compared to leaf signifying their importance in plant development too. Evaluation of the expression of this gene family in field grown tissues highlighted the prominent role they have in providing thermo-tolerance during daily temperature variations. The function of three putative sHSPs was established as holdase chaperones as evidenced by protection to malate-dehydrogenase against heat induced protein-aggregation. This study provides insights into the characterization of the Acd genes in tomato and forms the basis for further functional validation *in-planta*.

## Introduction

The α-crystallin domain (ACD) is an evolutionarily conserved domain from archaea to eukaryotes (Bondino et al., 2012). The name ACD is derived from the eye lens α-crystallin protein, a chaperone preventing non-native or denatured proteins from aggregation and preventing cataracts in vertebrates (Horwitz, 1992). This domain comprises of two conserved regions that form a sandwich of two pleated β-sheets separated by a hydrophilic domain of variable length. There are four main groups of the ACD proteins viz. the small heat shock proteins (sHSPs) group, the transcription factor (TF) group, sodium-lithium (NaLi) group, and the uncharacterized ACD proteins (UAP) group (Bondino et al., 2012).

ACD proteins are known to perform different functions, such as (i) transcriptional regulation (Zhu et al., 2008), (ii) protection from sodium and lithium salt stress (Matsumoto et al., 2001), and (iii) in inhibition of virus movement through the phloem (Whitham et al., 2000). The ACD protein, REPRESSOR OF SILENCING5 (ROS5), is required for DNA demethylation in *Arabidopsis thaliana* (Zhao et al., 2014). Moreover, plant sHSPs (having molecular weight of ~15–42 kDa) protect cells against deleterious effects of a wide array of environmental cues including not only heat but also low temperature, dehydration, high light intensity, UV, osmotic stress, oxidative stress, and also participate in plant-pathogen interaction (Vierling, 1991; Nover and Scharf, 1997; Sun et al., 2002; Zhao et al., 2007; Merino et al., 2014). The N-terminus of sHSP participates in binding to denatured proteins (Jaya et al., 2009), whereas the C-terminus is involved in homo-oligomerization (Giese and Vierling, 2004) and the formation of heat stress granule (Kirschner et al., 2000), the site for transiently storing denatured protein-sHSP oligomers during heat stress. The sHSPs have also been reported to regulate the activity heat shock factors (Hsfs) by influencing the transcriptional activity, solubility and intracellular distribution of Hsfs to modulate thermo-tolerance during heat stress (Scharf et al., 1998; Port et al., 2004).

Constitutive over-expression of *Castanea sativa* (chestnut) *HSP17.5* has been shown to substantially enhance the basal thermo-tolerance of hybrid poplar without affecting plantation yields (Merino et al., 2014). Expression of sHsp genes has also been associated with pollen development, pectin depolymerization and juice viscosity in ripening fruits and seed and/or fruit development in plants (Sun et al., 2002; Ramakrishna et al., 2003; Neta-Sharir et al., 2005; Giorno et al., 2010).

Tomato (*Solanum lycopersicum*) is a major fruit crop with 4.80 million hectares under cultivation world-wide (FAOSTAT 2012; www.faostat.fao.org/default.aspx). Most tomato cultivars are affected by abiotic stresses, resulting in reduced crop quality and yield (Kadyrzhanova et al., 1998; Giorno et al., 2010). The identification and classification of ACD proteins in plants is still in its infancy, moreover, a comprehensive characterization of Acd genes in tomato is lacking. Only few attempts have been made to understand the structural and functional aspects of some tomato sHsp genes; these have been marked in Table 1. In *A. thaliana* 44 Acd genes are found; of these 19 genes code for sHSPs, while in *Oryza sativa* 43 Acd genes are reported, of these 24 encode sHSPs (Bondino et al., 2012). In this study, we have identified 50 Acd genes in tomato using *in-silico* approaches. Both tandem and segmental duplications events appear to have shaped the expansion of this gene family in tomato leading to functional diversification. Expression analysis of selected genes using quantitative polymerase chain reaction (qPCR) and publicly available microarray data in response to various abiotic stresses as well as different phytohormones reveals cross-talk between stress- and hormone-inducible pathways in regulating the expression of Acd genes. Some of these genes are also involved in plant development, with high expression in roots and in reproductive tissues. Further, in order to understand the response of Acd genes in nature, expression profiling was done in different plant organs and in response to diurnal variations using field grown tissues during the month of May, when plants experience temperatures above 40°C during daytime. The mode of action of three putative tomato sHSPs as chaperones was established *in-vitro*.

**Table 1:** **Nomenclature and feature list of 50 ACD containing protein identified in tomato**.

**Class**	**Gene name**	**Locus**	**Best hit SGN-unigene no**.	**ORF (aa)**	**Mol wt (kDa)**	**Intron**
CI sHSP	*SlHsp15.6-CI*	Solyc02g093600.2	SGN-U565386	136	15.6	1
	*SlHsp17.7A-CI*^a^	Solyc06g076520.1	SGN-U578930	154	17.7	0
	*SlHsp17.6A-CI*^b^	Solyc06g076540.1	SGN-U580742	154	17.6	0
	*SlHsp17.6B-CI*^b^	Solyc06g076570.1	SGN-U578203	154	17.6	0
	*SlHsp17.6C-CI*^b^	Solyc06g076560.1	SGN-U578134	154	17.6	0
	*SlHsp9.1-CI*	Solyc07g045610.1	No significant score	78	9.1	1
	*SlHsp24.5-CI*	Solyc09g011710.2	SGN-U566729	208	24.5	1
	*SlHsp15.2-CI*	Solyc09g015000.2	SGN-U590520	134	15.2	1
	*SlHsp17.7B-CI*	Solyc09g015020.1	SGN-U580005	154	17.7	0
	*SlHsp27.1-CI*	Solyc10g086680.1	SGN-U574789	234	27.1	0
CII sHSP	*SlHsp17.3-CII*^c^	Solyc08g062340.2	SGN-U579132	155	17.3	0
	*SlHsp17.6-CII*^d^	Solyc08g062450.1	SGN-U581229	158	17.6	0
CIII sHSP	*SlHsp16.1-CIII*^e^	Solyc03g123540.2	SGN-U575804	144	16.1	1
ER sHSP	*SlHsp21.6-ER*	Solyc01g102960.2	SGN-U581282	189	21.6	0
	*SlHsp21.5A-ER*	Solyc03g113930.1	SGN-U578245	188	21.5	0
	*SlHsp21.5B-ER*^f^	Solyc11g020330.1	SGN-U573948	190	21.5	0
MTI sHSP	*SlHsp23.8-MTI*^g^	Solyc08g078700.2	SGN-U578546	210	23.8	1
P sHSP	*SlHsp26.2-P*^h^^,^ ^i^	Solyc03g082420.2	SGN-U581239	235	26.2	1
	*SlHsp25.7-P*^j^	Solyc05g014280.2	SGN-U568478	221	25.7	2
	*SlHsp21.5-P*	Solyc08g078710.1	Not found	196	21.5	1
PX sHSP	*SlHsp16.1-PX*	Solyc04g014480.2	SGN-U581793	145	16.1	1
	*SlHsp26.5-PX*	Solyc07g055720.2	SGN-U590141	238	26.5	5
NaLi	*SlAcd57.6-NaLi*	Solyc04g011460.1	SGN-U586331	508	57.6	0
	*SlAcd57.1-NaLi*	Solyc10g086420.1	SGN-U575646	500	57.1	0
	*SlAcd58.0-NaLi*	Solyc11g066090.1	SGN-U568501	511	58.0	0
TF	*SlAcd61.8-TF*	Solyc04g082820.2	SGN-U583264	561	61.8	12
	*SlAcd48.0-TF*	Solyc12g094730.1	No significant score	422	48.0	11
UAP I	*SlAcd15.7-CI*	Solyc02g080410.2	SGN-U585356	137	15.7	1
UAP III	*SlAcd17.9-CIII*	Solyc04g072250.2	SGN-U575857	163	17.9	1
UAP IV	*SlAcd21.6-CIV*	Solyc07g064020.2	SGN-U579132	188	21.6	1
UAP V	*SlAcd17.3-CV*	Solyc03g113170.1	No significant score	154	17.3	0
	*SlAcd23.8-CV*	Solyc03g113180.2	SGN-U598172	209	23.8	1
UAP VI	*SlAcd49.3-CVI*	Solyc01g096960.2	SGN-U572986	441	49.3	1
	*SlAcd39.4-CVI*	Solyc01g096980.1	SGN-U572985	348	39.4	1
UAP VII	*SlAcd25.7-CVII*	Solyc01g009200.2	No significant score	232	25.7	1
	*SlAcd23.8-CVII*	Solyc01g009220.2	Not found	213	23.8	1
	*SlAcd26.8-CVII*	Solyc09g007140.2	SGN-U566621	236	26.8	1
	*SlAcd15.5-CVII*	Solyc10g076880.1	Not found	139	15.5	1
	*SlAcd27.6-CVII*	Solyc11g071560.1	SGN-U566621	247	27.6	1
UAP VIII	*SlAcd37.0-CVIII*	Solyc04g071490.2	Not found	325	37.0	1
	*SlAcd27.2-CVIII*	Solyc12g056560.1	No significant score	240	27.2	1
UAP IX	*SlAcd32.3-CIX*	Solyc03g005190.2	SGN-U581239	293	32.3	1
	*SlAcd23.1-CIX*	Solyc06g054150.1	SGN-U568281	205	23.1	0
	*SlAcd16.0-CIX*	Solyc06g084220.1	SGN-U581986	145	16.0	0
	*SlAcd18.0-CIX*	Solyc09g065370.1	SGN-U581646	162	18.0	0
	*SlAcd11.3-CIX*	Solyc09g082150.1	SGN-U576030	109	11.3	0
UAP X	*SlAcd54.0-CX*	Solyc01g098790.1	SGN-U601615	487	54.0	1
	*SlAcd24.6-CX*	Solyc01g098810.2	SGN-U570604	230	24.6	2
	*SlAcd16.7-CX*	Solyc04g082720.2	SGN-U578542	153	16.7	1
	*SlAcd21.6-CX*	Solyc04g082740.2	SGN-U562947	197	21.6	1

Characterized in

a *Fray et al. (1990)*,

b *Goyal et al. (2012)*,

c *Giorno et al. (2010)*,

d *Kadyrzhanova et al. (1998)*,

e *Siddique et al. (2003)*,

f *Zhao et al. (2007)*,

g *Liu and Shono (1999)*,

h *Lawrence et al. (1997)*,

i *Neta-Sharir et al. (2005)*,

j *Ramakrishna et al. (2003)*.

*ORF, open reading frame*.

## Materials and methods

### Plant material and stress conditions

Tomato (cultivar Pusa Ruby) seeds were procured from the Indian Agricultural Research Institute, New Delhi, India. Seeds were germinated at 26°C. Three-days-old seedlings of uniform growth were transplanted in plastic pots, filled with soilrite (horticulture grade expanded perlite, Irish Peat moss, and exfoliated vermiculite in equal ratio i.e., 1/3:1/3:1/3) and placed in a plant growth chamber (CMP6050, Conviron, Canada) maintained at 26/21°C (day/night: 16/8 h) relative humidity 60%, light intensity 300 μmol per m^2^ per sec. Thirty-days-old plants were subjected to all the stresses/treatments.

Heat stress consisted of the following regimes: Basal heat stress: Plants were directly exposed for 2 and 4 h at 45°C. Acclimated heat stress: Plants were initially exposed for 2 h at 35°C, then recovered overnight in a chamber at 26/21°C (day/night: 16/8 h) followed by exposure for 2 and 4 h at 45°C. In addition, plants from all the above regimes were allowed to recover overnight in a chamber maintained at 26/21°C (day/night: 16/8 h). For, low temperature stress, plants were placed at 4 ± 2°C for 2 days. Dehydration stress was imposed by withholding water for 12 days when the leaf relative water content (RWC) was ~60% (data not shown). RWC was measured as described by Muoki et al. (2012a). For salt stress treatment, a set of plants were uprooted carefully and transferred to half strength Murashige and Scoog (MS) medium and stabilized for 2 days. The plants were then transferred to fresh half strength MS solution containing 170 mM sodium chloride. MS solution without sodium chloride, served as control. Plants were given chemical treatments by spraying 0.1 mM salicylic acid (SA; Sigma, USA), 5 mM hydrogen peroxide (Merck, Germany), and 100 μM abscisic acid (ABA; Sigma, USA) three times for 24 h. The concentrations of solutions used were chosen based on previous reports (Hua et al., 2009; Sarkar et al., 2009). Control plants were treated with water. Each experiment was repeated two times with separate biological materials to ensure reproducibility of data. Leaves from these experimental plants were harvested at relevant time points frozen in liquid nitrogen and kept at −80°C until use.

In a separate experiment, leaf tissues from 4 month old field-grown tomato bushes in the month of May were collected between 08:00 and 09:00 h (morning), 14:00 and 15:00 h (afternoon), and 17:00 and 18:00 h (evening) for 2 consecutive days. Temperature and relative humidity were measured using an electronic digital hygro-thermometer (Fisher Scientific, USA). Light intensity was recorded using a digital lux meter (TES 1332, TES Electrical Electronic Corp., Taiwan). Root, flower, and fruit (mature-green and red-ripe) tissues were also collected between 08:00 and 09:00 am.

### Identification of Acd genes and sequence analyses

Data mining using HMMER profiling was used to obtain ACD proteins in tomato. ACD proteins sequences from 17 plant species were collected and grouped into classes according to existing nomenclature for ACD proteins (Bondino et al., 2012). For each class, multiple sequence alignments were generated using ClustalX 2.1 (clustalx.software.informer.com/2.1/) and ACD-specific Hidden Markov Model (HMM) profiles were generated with these alignments as in-put. These in-house generated HMMs were used to retrieve ACD proteins from the proteome of tomato (ITAG release 2.3; http://sgn.cornell.edu). Hits with *e* < e^−10^ were taken.

Domain searches were performed using batch search tool at the conserved domain database (CDD) at National Center for Biotechnology Information (NCBI) (http://www.ncbi.nlm.nih.gov/Structure/cdd/cddsrv.cgi). Molecular weights were calculated by Prot param (http://web.expasy.org/protparam/). For *in-silico* analysis of putative promoter sequences, the 2 kb sequences upstream of the translational start codon (ATG) of each Acd gene was downloaded from Sol Genomics Network (SGN) database and analyzed for the presence of putative *cis*-regulatory elements by PlantCARE (Rombauts et al., 1999). Gene Ontology (GO) terms were searched at SGN database for selected genes. Putative interacting protein partners was predicted using an *in-silico* protein-protein interaction software STRING (http://string-db.org/).

### Phylogeny, chromosomal localization, and duplication events

Neighbor-Joining phylogenetic trees were generated on full length amino acid sequences using ClustalX 2.1 with default settings and the bootstrap test carried out with 1000 iterations. The resultant tree was visualized with TreeView (version 1.6.6, http://taxonomy.zoology.gla.ac.uk/rod/treeview.html). The location of the Acd genes on tomato chromosomes was determined using the BLASTN search tool at SGN database. The resulting positions of these genes were mapped on the tomato chromosomes using Mapchart 2.2 (Voorrips, 2002). Paralogs were detected using PLAZA Tool (http://bioinformatics.psb.ugent.be/plaza/) available at Plant Genome Duplication Database that makes use of integrative orthology tools. The hits were further confirmed using phylogeny with gene-pairs grouping at more than 80% bootstrap value. The number of non-synonymous substitutions per non-synonymous site (*Ka*) and synonymous substitution per synonymous site (*Ks*) values of the paralogous genes were estimated using DnaSPv5.0 (Librado and Rozas, 2009). The *Ks* values were then used to calculate the approximate date of the duplication event (*T* = *Ks*/2λ), assuming clock-like rates (λ) of synonymous substitution of 1.5 × 10^−8^ substitutions/synonymous site/year for tomato (Blanc and Wolfe, 2004).

### Quantitative PCR analysis

Total RNA was isolated from leaf tissues using Ribozol (Ameresco, USA), whereas from flower, fruit and root, RNA was isolated as described by Muoki et al. (2012b). DNase I (Ambion, USA) treated total RNA was used to synthesize cDNA using high capacity cDNA reverse transcription kit (Applied Biosystem, USA) following the manufacturer's protocol. Gene expression was performed on 7900 HT fast real time PCR system under default settings (Applied Biosystem, USA) using 2 × Brilliant III SYBR^@^ Green qPCR Master Mix (Agilent Technologies, USA) and gene specific primers (Supplementary Table 1A). All qPCRs were run in duplicates, each having three technical replicates, with a no-template control to check for contamination. Positive controls for each stress and tissue were included based on published literature; see details about selected genes, primers used, and references in Supplementary Table 1B). Gene expression was calculated by the 2^−ΔCt^ and 2^−ΔΔCt^ methods using the Relative Expression Software Tool (REST; Pfaffl et al., 2002) and the housekeeping gene *Actin*. Expression profiles and heat maps were generated by using Multi-Experiment Viewer software (Saeed et al., 2003). Expression data was statistically analyzed using the software Statistica 13.0 (Stat Soft. Inc. USA) for analysis of variance (ANOVA) to identify genes expressing at >two-fold in different stages of development as well as various stress conditions, with *P* < 0.05. Duncan test was used for distinguishing the mean differences which were significantly different.

### Microarray-based expression analysis

The microarray data-based expression profiles for different organs and abiotic stresses including heat, dehydration, and salt stress condition was assessed using meta-analysis tool at Genevestigator (http://www.genevestigator.ethz.ch) on Le_10K microarray platform. The genes exhibiting at least two-folds change and above with *p* < 0.05 were considered as significant. The expression was computed in form of heat maps for abiotic stress conditions.

### Recombinant proteins expression and holdase chaperone assay

The open reading frame (ORF) sequences for *SlHsp17.6C-CI, SlHsp24.5-CI, SlHsp26.5-PX*, and *SlAcd15.7-CI* were amplified using cDNA synthesized from total RNA isolated from acclimated high temperature stressed tomato leaf tissue (4 h), cloned into *Sma*I linearized pUC19 (Thermo Scientific, USA) and confirmed by sequencing. The ORFs were then introduced into the *pET28a* vector between the *Nde*I and *Xho*I sites for recombinant protein expression. The gene specific primers used for ORF cloning are listed in Supplementary Table 1C. The *pET28a-His-Hsp17.6C-CI, pET28a-His-Hsp24.5-CI, pET28a-His-Hsp26.5-PX*, and *pET28a-His-Acd15.7-CI* plasmids were transformed into *E. coli* BL21, BL21 (DE3), BL21 (DE3) pLys, and BL21 Rosetta cells, respectively. Recombinant proteins were expressed, purified and holdase chaperone activity was measured as described previously (Paul et al., 2014; Li et al., 2015). Briefly, protein expression was induced at 37°C with 0.1 mM isopropyl β-D-1-thiogalactopyranoside for 4 h. The recombinant protein was purified using Ni-NTA affinity chromatography (Thermo scientific, USA) according to the manufacturer's protocol. The resulting proteins were separated using SDS-PAGE (Supplementary Figure 1). Recombinant proteins were dialyzed against 50 mM Tris-HCl (pH 8.0) overnight. The dialyzed proteins were quantified with Bradford reagent (Sigma, USA) using bovine serum albumin (BSA) as standard. The protein capacity to suppress thermal aggregation of porcine malate dehydrogenase (MDH; Sigma, USA) was used to measure its holdase chaperone activity. The reaction buffer (40 mM HEPES, pH 7.5) containing protein samples were heated in a water bath maintained at 45°C for up to 40 min. MDH aggregation was monitored by measuring absorbance at 340 nm using a spectrophotometer (NanoDrop 2000c; Thermo scientific, USA). Each assay set was repeated at least two times. In each set, recombinant His-SlAcds were prepared independently.

## Results and discussion

### Fifty genes encode for ACD proteins in tomato

In order to obtain ACD proteins in tomato, rigorous data mining was performed using HMMER. From a repertoire of published sequences belonging to different classes of ACD proteins (Bondino et al., 2012), specific HMM profiles were built in-house for each class. Fifty putative ACD proteins were identified (Table 1) in tomato that were further confirmed for the presence of the ACD in CDD database at NCBI (Supplementary Table 2). These sequences were categorized into four groups having a total of 18 classes and named essentially as described by Bondino et al. (2012). The largest number of ACD proteins set (23) belongs to the poorly annotated UAP group (Bondino et al., 2012) that has nine classes (Class-I and Class-III to -X) within it (Table 1). The second largest group includes 22 members, belonging to the classical sHSP group that has been extensively annotated in literature (Siddique et al., 2008). These sHSPs were further categorized into the previously described eight classes viz., Class-I, -II, -III, plastidial (P), mitochondrial (MT), endoplasmic reticulum (ER), and peroxisomal (PX) (Table 1). Of the remaining five ACD proteins, two belong to the TF group and three to the NaLi group. Similar to the other plant TF class of ACD proteins (Bondino et al., 2012), the tomato TF members also have an AT-rich interaction domain (ARID; Supplementary Table 2) involved in transcriptional regulation (Zhu et al., 2008). NaLi proteins have a putative uncharacterized N-terminal domain (Bondino et al., 2012) and are suggested to be involved in sodium and lithium salt stress (Matsumoto et al., 2001). In addition to the conserved ACD; yeast transposon protein A (TYA) and histone H1-like nucleoprotein (HC2) domains involved in DNA condensation are present in SlAcd49.3-CVI. The domain of unknown function (DUF966) and 2Fe-2S iron-sulfur cluster binding (Fer2) domain involved in electron transfer processes and various enzymatic reactions are found in SlAcd26.8-CVII and SlAcd32.3-CIX, respectively (Supplementary Table 2). Gene Ontology (GO) terms were searched to further assign putative functions to the 23 UAP genes (Supplementary Table 3). The analysis suggested SlAcd17.9-CIII possesses protein homo-dimerization activity, whereas SlAcd23.1-CIX, SlAcd16.0-CIX, and SlAcd18.0-CIX have protein binding functions. Like the CDD search, the GO term for SlAcd32.3-CIX also suggested its role in electron transport chain process. GO terms were not found for the remaining 18 UAP genes.

We find that some of the ACD classes are lacking in tomato, these include 2 sHSP classes, -MTII and -plastidial-like (P-like) and 2 UAP classes, -II and -XI. Although Bondino et al. (2012) have reported one MTII sHSP member in tomato, the reason why we did not find it in our study is because the protein sequence is absent in the current SGN proteome, though a corresponding SGN unigene (SGN-U563517) is available. We further note that some ACD protein classes are missing in other species too. *A. thaliana* is deficient in sHSP class-P-like and UAP classes -II, -III, and -V, while UAP classes -III, -VI, -VIII, -X, and -XI are absent in *O. sativa* (Supplementary Table 4). A study comprising 824 ACD proteins from 17 plants (including 13 dicot and 4 monocot species) showed that sHSP class-P-like is present only in monocots, whereas UAP class-XI proteins, that is characterized by the presence of more than 1 ACD in them, is present in only some dicots like *A. thaliana, A. lyrata*, and *Ricinus communis*. Further, UAP class-VI and -VIII are absent in monocots (Bondino et al., 2012). Clearly during the course of evolution, this gene family has experienced conservation, loss as well as expansion of its members in the two angiosperm lineages: monocot and dicot.

### Evolutionary relatedness between plant ACD proteins

**Figure 1:**
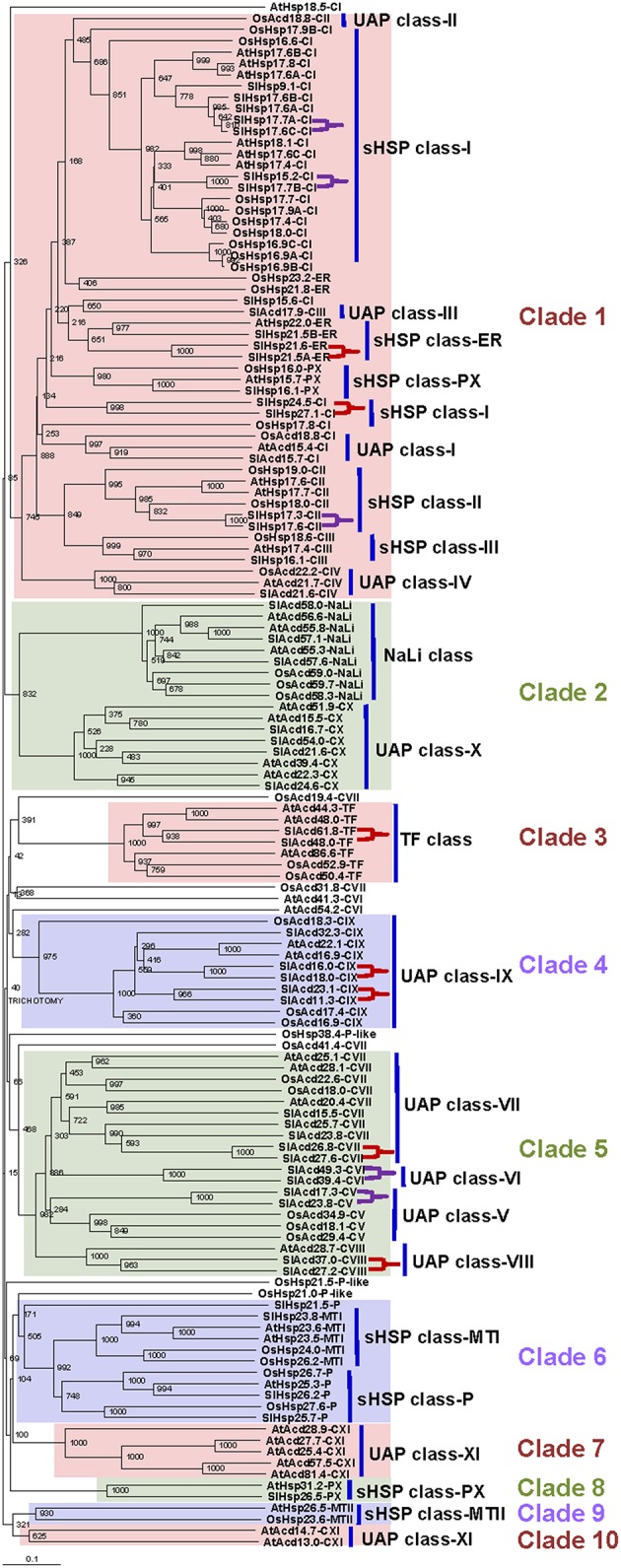
**Phylogenetic analysis of tomato Acd gene family**. The tree was derived by Neighbor-joining method with bootstrap analysis (1000 replicates) from alignment of ACD proteins of tomato, *A. thaliana* and *O. sativa* using ClustalX2.1. The tree was analyzed with TreeView 1.6.6. Numbers at the nodes denote bootstrap values. Tree branches with bootstrap value above 50% were delineated to represent a clade. The different clades are highlighted and numbered 1–10. The sub-clades representing different ACD proteins have being marked by bold vertical lines. The paralogous pairs are marked by purple and red brackets for tandem and segmental duplication events, respectively. The genetic distances are indicated by the horizontal bar.

Multiple sequence alignment was generated using the tomato, *A. thaliana* (another dicot) and *O. sativa* (a monocot) ACD protein sequences (Siddique et al., 2008; Sarkar et al., 2009; Bondino et al., 2012) to gain insights into the evolutionary relationship of this gene family in plants (Figure 1). Keeping a bootstrap value above 50% as a yard stick to delineate clades, 10 distinct groups could be defined. The phylogenetic tree reveals that clade 1 consists of the classical sHSP classes viz., Class-I, -II, -III, -ER, -PX, represented by 17 tomato sHSPs, and also some members from four UAP classes namely class-I, -II, -III, and -IV. This is in accordance with prior reports; Siddique et al. (2008) had classified UAP class-I proteins as sHSP class-V in *A. thaliana*. Similarly, Bondino et al. (2012) had shown UAP class-I, -II, and -III to group together with sHSPs suggesting their close proximity. Four other tomato sHSPs are present in clade 6 having two distinct sub-clades: P and MTI. It is interesting to note that while two tomato members of the sHSP class-P group with the *A. thaliana* and *O. sativa* members, SlHsp21.5-P appears to have diverged independently. The last tomato sHSP groups with its *A. thaliana* counterpart in clade 8 representing sHSP class-PX. Clade 9 consists of sHSPs class-MTII, whose members are absent in tomato. The other clades include members of the remaining ACD proteins. Clade 2 includes NaLi and UAP class-X sub-clades. Clades 3 and 4 consist of TF and UAP class-IX, respectively; while clade 5 includes UAP class-V, -VI, -VII, and -VIII. Only *A. thaliana* has members of UAP class-XI, these proteins group into two separate clades- clade 7 and 10. Several clades represented members from all the three organisms signifying a common ancestry and similar course of evolutionary path. Many classes like ER sHSP (clade 1) and NaLi (clade 2) have distinct monocot- and dicot-specific sub-clades. UAP class-X (clades 2), class-VIII (clade 5), class-XI, (clade 7 and 10), and sHSP class-PX (clade 8) are restricted to dicots only while members of P-like are present in only *O. sativa* and do not form any specific clade of their own. This suggests that while most members have been conserved during the course of evolution, lineage-specific functional diversification of some ACD protein has occurred that expanded the scope of the roles of the ACD proteins in plants during evolution.

### Tomato Acd genes are widely distributed in the genome with 12 gene pairs exhibiting duplication events

To determine the genomic distribution of the tomato Acd genes, their chromosomal locations were analyzed. Results show that the 50 Acd genes are dispersed on all 12 tomato chromosomes (Supplementary Figure 2). Chromosome 1 and 4 have the maximum of seven genes each, while six genes each are found on chromosome 3, 6, and 9, four genes are on chromosome 8, three each are on chromosome 7, 10, and 11, two each are on chromosome 2 and 12, and one is on chromosome 5. Gene duplication by way of segmental and/or tandem duplication events forms the key source for the genesis of new genes, which in turn facilitates generation of novel functions (Hurst, 2002). It is believed that segmentally duplicated genes are more often retained in the more slowly-evolving gene families (like MYB transcription factors family) whereas, in the rapidly-evolving families (like the ones related to plant defense e.g., NBS-LRR), duplication in local genomic clusters (tandem duplication) is common (Cannon et al., 2004). It has been established that the Solanum lineage has experienced two consecutive genome triplications that formed the basis for the neofunctionalization of genes (The Tomato Genome Consortium, 2012). Our analysis identifies 12 pairs of paralogous Acd genes in tomato (Supplementary Figure 2; Supplementary Table 5). Paralogs were initially inferred using PLAZA database that utilizes integrative orthology methodology (Best-Hits-and-Inparalogs). These hits were further confirmed using their proximity in a phylogenetic tree at a bootstrap value of >80% (Figure 1; marked by brackets). Seven pairs (58%) of the paralogous genes (including 4 UAP, 1 TF, and 2 sHSP) are randomly scattered throughout the genome, suggesting putative segmental duplication events. Earlier, utilizing 50 diverse gene families, Cannon et al. (2004) has shown that gene families having diverse enzymatic functions tend to have medium to high tandem duplications; the Acd gene family definitely qualifies as one such family. The remaining 5 paralogous pairs (~42%; belonging to 3 sHSP and 2 UAP classes) are located in close proximity at the chromosome level, which probably resulted from tandem duplications (Supplementary Figure 2). We note that all the six Acd genes on chromosome 9 are members of paralogs, belonging to both tandem and segmental duplication events. On the other hand, no paralogs are present on chromosome 2, 5, and 7.

The synonymous substitution rates (Ks) and non-synonymous substitution rates (Ka) are measures to explore the gene divergence mechanism after duplication. Under the assumption that synonymous changes approximate the neutral rate of molecular evolution, a Ka/Ks value significantly above 1 (or Ka > Ks) provides evidence for positive selection for amino acid substitution. In contrast, a Ka/Ks below 1 (or Ka < Ks) suggests a purifying selection (Hurst, 2002). The results for tomato paralogous Acd gene pairs shows that the Ka/Ks ratios for only one duplicated pair is >1, whereas the Ka/Ks ratios for the remaining 11 pairs are < 1 (Supplementary Table 6). This suggests that owing to the important roles the Acd gene family members play in plant survival, the duplicated Acd genes are more constrained and under purifying selection pressure. Overall, the gene duplication pattern indicated that segmental duplication and tandem duplication together might contribute to the expansion of Acd genes in tomato with the gene pairs under strong conservation pressure.

### Differential expression of Acd genes under varied heat stress regimes

**Figure 2:**
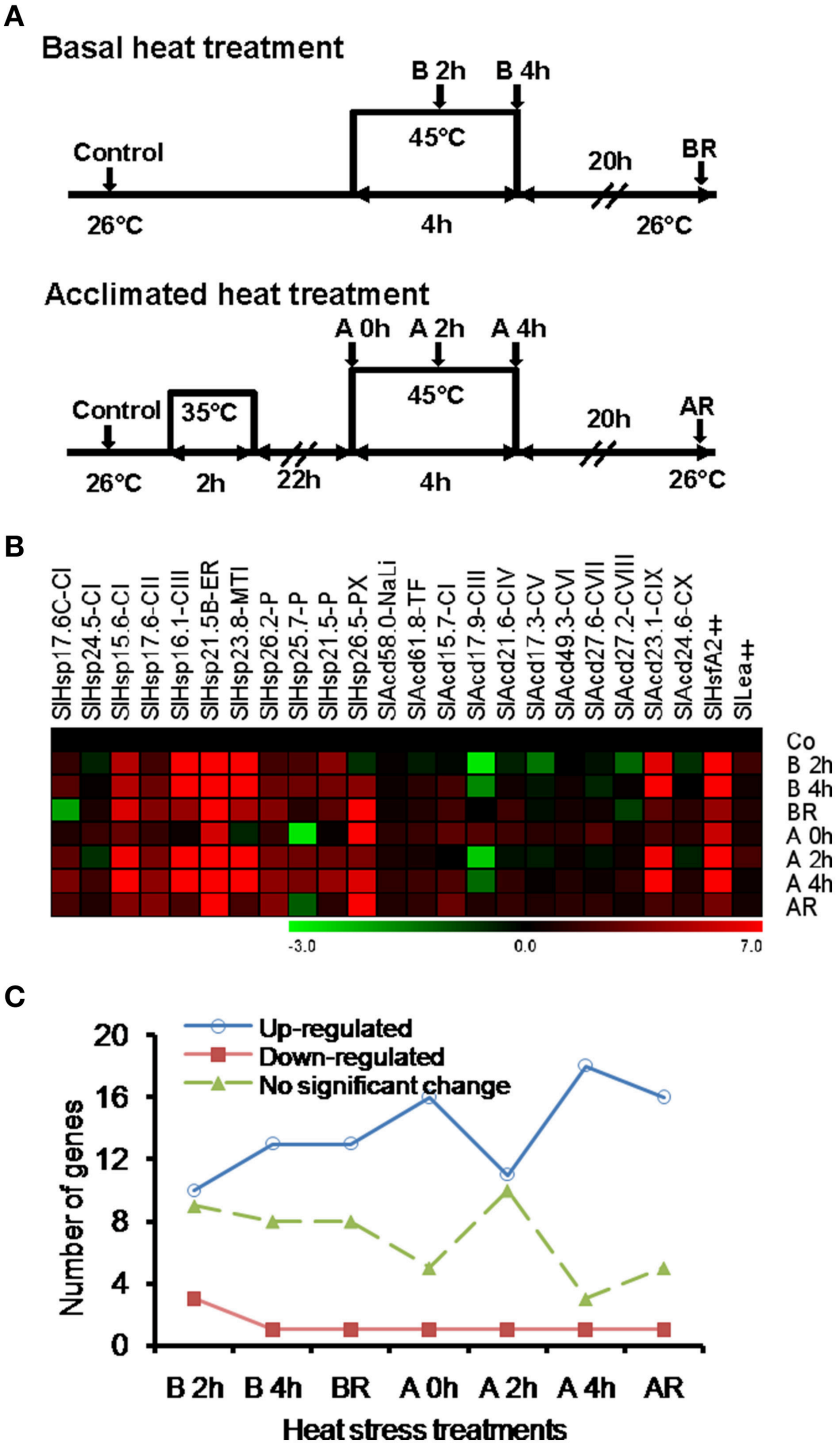
**Heat treatment and gene expression analysis. (A)** Diagrammatic representation of the heat treatment regimes. Shaded arrows represent sampling times and their designations. **(B)** Heat map of tomato Acd expression in response to different heat treatment regimes. **(C)** Graphical depiction of number of genes modulated by different heat treatment regimes. The expression levels of genes were calculated using the 2^−ΔΔCt^ method and presented using fold change values transformed to log 2 relative to control. Transcriptional change values, as calculated by relative expression software tool (REST), along with details of ANOVA and standard error are shown in Supplementary Table 7A. Green and red colors indicate down- and up-regulation of the genes, respectively. Control (Co), basal 2h (B 2h), basal 4h (B 4h), basal recovery (BR), acclimated 0h (A 0h), acclimated 2h (A 2h), acclimated 4h (A 4h), acclimated recovery (AR). “‡” Symbol represents control genes obtained from previous publication (see Supplementary Table 1B for detailed information).

Prior exposure to a short, sub-lethal temperature rapidly acclimates plants to normally lethal high temperatures, a phenomenon known as acquired thermo-tolerance (Vierling, 1991; Sun et al., 2002). This elicits significant diversity in transcripts at cellular level than when subjected directly to severe heat stress (Larkindale and Vierling, 2008). Studies have indicated that sHSPs are also associated with thermo-tolerance (Zhao et al., 2007; Hua et al., 2009; Giorno et al., 2010; Ruibal et al., 2013; Merino et al., 2014). To get insights into the expression profiling of the sHsp and other Acd family members under different heat stress regimes, gene expression analysis was performed in response to heat stress (45°C) without acclimation (basal heat stress) or following acclimation treatment as shown in Figure 2A (See Section Materials and Methods for details). We analyzed expression of 22 (representing all the classes; three from sHSP-CI and -P family and one each from the 16 remaining families) genes randomly selected using qPCR. Only genes with expression level of two-folds or more were considered significant. Further, statistical significance was determined by ANOVA with *p* < 0.05.

Seventeen genes including the 11 classical sHsps, 4 UAP (*SlAcd15.7-CI, SlAcd21.6-CIV, SlAcd27.2-CVIII*, and *SlAcd23.1-CIX*) and one each of NaLi (*SlAcd58.0-NaLi*) and TF (*SlAcd61.8-TF*) class are up-regulated by heat stress at 45°C for 4 h either with/without acclimation (Figure 2B; Supplementary Table 7A). We note that more genes get up-regulated when the duration of heat exposure increases from 2 to 4 h in both the regimes (Figure 2C) signifying that it is not purely the degree of heat stress but also the duration that is important for significant transcript accumulation. We find that for most of these up-regulated genes, there is no significant difference in the level of expression in response to the two stress regimes (Figure 2B; Supplementary Table 7A), signifying a common response elicited by the plants in response to heat stress. However, some differences were also noted. More genes are up-regulated in all acclimation stages (including recovery) than in basal heat stress (Figure 2C). The expression is significantly higher (>two-folds) in acclimated tissues as compared to basal tissues exposed to 45°C for 4 h in *SlHsp17.6C-CI, SlHsp24.5-CI, SlHsp15.6-CI, SlHsp26.5-PX*, and *SlAcd27.2-CVIII* (Supplementary Table 7A). These observations regarding differential response of genes to variable stress levels are in accordance with previous reports in *A. thaliana* (Larkindale and Vierling, 2008) that established that some transcripts are specific to basal heat tolerance, while others are involved during the preconditioning of plants to acquire thermo-tolerance, and many are common to both.

Only *SlAcd17.9-CIII* exhibits down-regulation at 45°C in both basal and acclimated tissues. In rice, it has been shown that the expression of Acd genes is either unaffected or down-regulated under heat stress; the exceptions being *OsAcd21.0* and *OsAcd30.2* that show up-regulation (Sarkar et al., 2009).

When the expression of these tomato genes was assessed upon recovery, there was a significant decline in the expression of heat stress induced genes upon cessation of high temperature (Figures 2B,C) for most of the genes. Similar transcriptional changes with genes exhibiting up-regulation by heat stress but down-regulation by recovery have been reported for *Vitis vinifera* (Liu et al., 2012). On the other hand, we find that *SlHsp24.5-CI, SlHsp26.5-PX SlAcd15.7-CI, SlAcd17.9-III* SlAcd21.6-CIV SlAcd17.3-CV, and SlAcd24.6-CX show higher (>two-folds) transcript abundance upon recovery compared to 2 h heat stress for both basal and acclimation regimes (Supplementary Table 7A). Moreover, *SlHsp15.6-CI, SlHsp26.5-PX SlAcd17.9-III, SlAcd21.6-CIV*, and *SlAcd24*.6-CX exhibit higher expression in basal-heat stressed recovered tissues (BR) as compared to basal-heat stressed tissues (B 2h and 4h, Supplementary Table 7A). In *Physcomitrella patens, PpHsp16.4* has been shown to exhibit relatively high expression level even upon heat stress relief (Ruibal et al., 2013). Similarly, in *A. thaliana*, Larkindale and Vierling (2008) have observed higher-fold transcript changes for some genes during recovery in acclimated seedlings as compared to seedlings subjected to direct heat stress. These Acd genes appear to have a role in recovery after heat stress in tomato. It has been shown that in addition to preventing protein denaturation during heat stress as molecular chaperones (Liu and Shono, 1999), some ACDs/sHSPs also have roles in removing denatured/misfolded proteins post-heat stress by facilitating their delivery to cellular proteases (Vierling, 1991; Sun et al., 2002). Interestingly, though no major difference in transcript abundance is observed between the two heat regimes for *SlHsp15.6-CI, SlHsp16.1-CIII, SlHsp23.8-MTI, SlHsp25.7-P*, and *SlHsp21.5-P* genes, their transcripts are significantly higher in basal-recovered tissues as compared to acclimated-recovered tissues (Supplementary Table 7A). This might be a consequence of higher cellular damage during basal-heat stress which in turn warrants more chaperonic activity for protection. Moreover, it is well known that the sHsp mRNAs are quite stable with half-lives of 30–50 h (Sun et al., 2002). SlHsp16.1-CIII, SlHsp23.8-MTI, and SlHsp25.7-P localize in the nucleus, mitochondria and chloroplast, respectively, and the latter two are reported to exhibit chaperone activity (Liu and Shono, 1999; Ramakrishna et al., 2003; Siddique et al., 2003). Thus, the data suggest that Acd genes play a pivotal role in heat stress tolerance and recovery processes with the mode of heat regimes influencing their expression pattern. The two regimes invoke transcriptional response via a common as well as unique set of genes.

**Figure 3:**
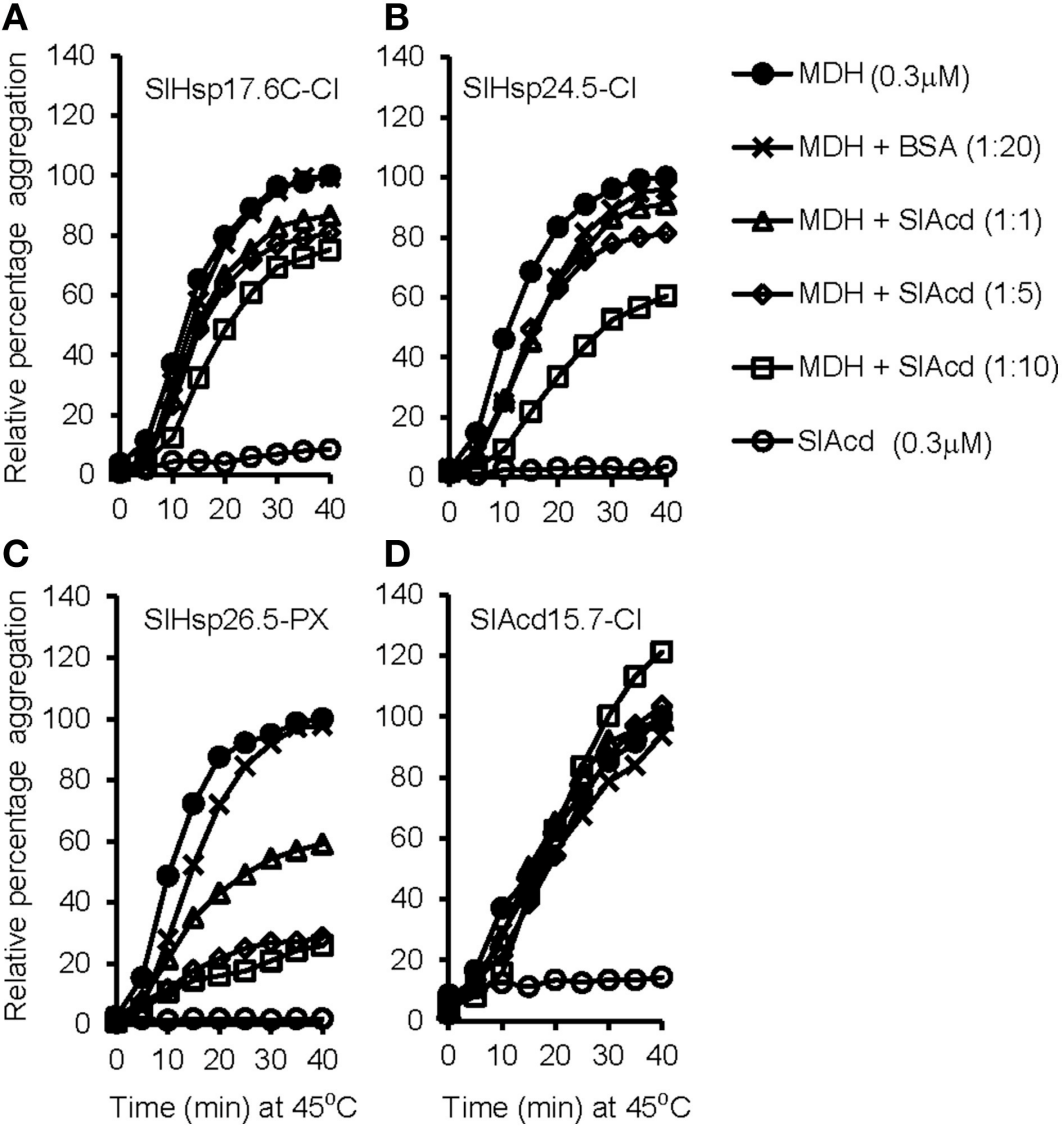
**Holdase chaperone activity of (A) SlHsp17.6C-CI; (B) SlHsp24.4-CI; (C) SlHsp26.5-PX; and (D) SlAcd15.7-CI with the thermo-labile substrate malate dehydrogenase (MDH)**. MDH (0.3 μM) was incubated with or without respective SlAcd at 45°C for the indicated time. Bovine serum albumin (BSA) was used as negative control. Each assay set was repeated for least two times with similar result and a representative graph is shown.

Under heat stress conditions, sHSPs prevent irreversible aggregation of proteins by binding to the exposed hydrophobic amino acids of partially denatured proteins. To determine if heat inducible SlAcds (Figure 2B) identified in the present study have chaperone activity that effectively maintain proteins in a folding-competent conformation, holdase chaperone activity of four putative tomato Acd proteins was studied. These include three previously uncharacterized sHSPs (SlHsp17.6C-CI, SlHsp24.5-CI, and SlHsp26.5-PX) and one UAP class-I Acd member, SlAcd15.7-CI that grouped together with other sHSPs in the phylogenetic tree (Figure 1). As shown in Figure 3, the thermo-labile porcine malate dehydrogenase (MDH) denatures upon heating at 45°C and aggregates, as monitored by light scattering at 340 nm over 40 min. However, in presence of SlHsp17.6C-CI, SlHsp24.5-CI, and SlHsp26.5-PX, heat-induced aggregation of MDH is protected in a dose-dependent manner (Figures 3A–C) confirming the mode of action of sHSPs as chaperones. However, SlAcd15.7-CI, did not exhibit any chaperone activity (Figure 3D). *In-silico* protein-protein interaction analysis (Supplementary Table 8) suggested SlAcd15.7-CI might function as an interacting partner of the Hsp70 chaperone complex to prevent protein denaturation (Liu and Shono, 1999) and/or with ATP-dependent Clp protease to remove denatured/misfolded proteins (Vierling, 1991; Sun et al., 2002).

### The Acd genes are responsive to various abiotic stresses and hormones

Plants being sessile are subjected to a variety of abiotic stresses, which hamper plant performance eliciting morphological, physiological, biochemical, and molecular changes. This leads to differential gene expression and increased modulation of several proteins, which are implicated in stress tolerance and survival. However, the mechanism varies depending upon the plant species, and also gene response under one cue may or may not be the same in response to other cues (Seki et al., 2002). Our study (Figure 2) finds heat inducibility of several Acd genes. Other stresses and hormones have also been reported to modulate the expression of this gene family (Siddique et al., 2008; Sarkar et al., 2009; Ruibal et al., 2013). This prompted us to investigate the cross-talk between heat stress and other environmental cues in this class of genes.

**Figure 4:**
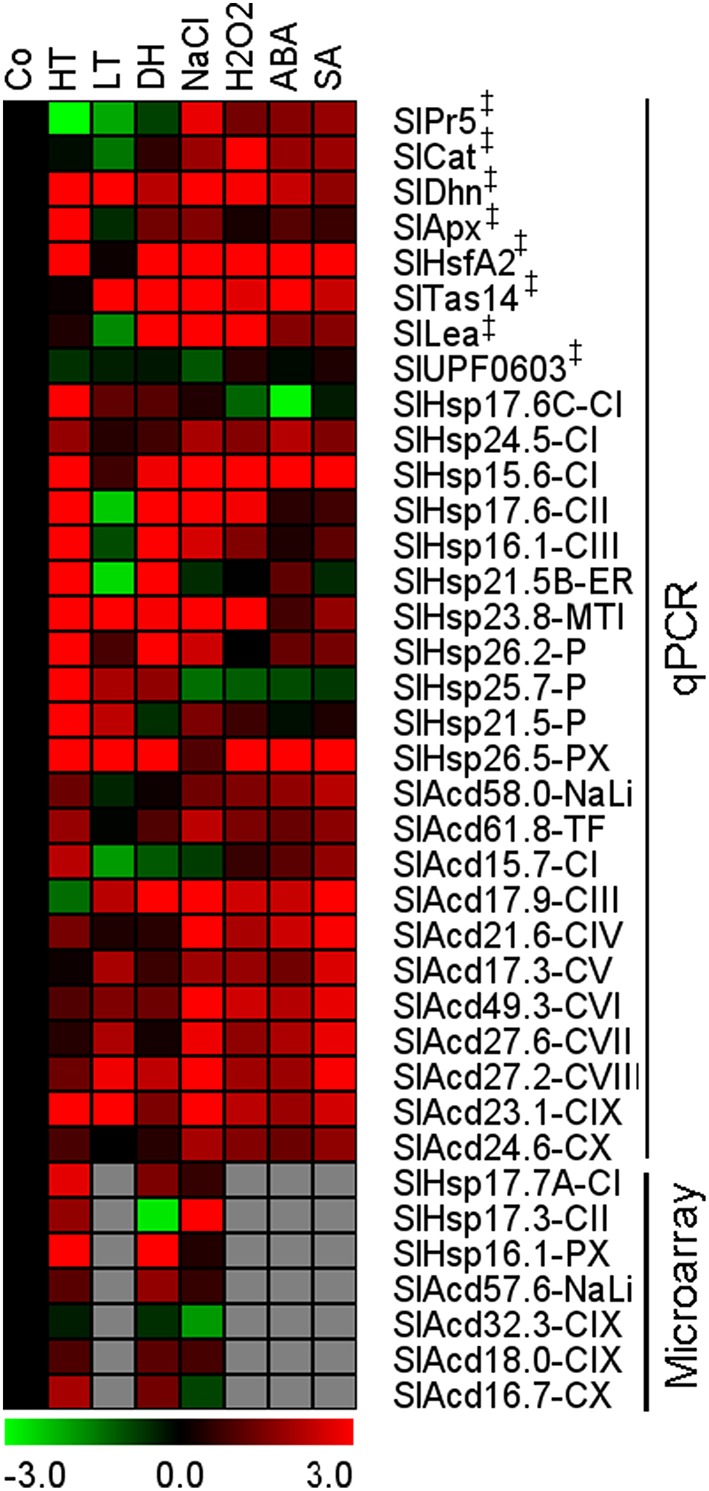
**Expression profiling of tomato Acd genes in various cues**. Heat map of Acd expression in response to heat (HT), dehydration (DH), low temperature (LT), salt (NaCl), abscisic acid (ABA), hydrogen peroxide (H_2_O_2_), and salicylic acid (SA) treatment. The micro-array based expression values (data available at Genevestigator) for 7 additional genes in response to HT, DH and salt were also combined. The expression levels of genes were calculated using the 2^−ΔΔCt^ method and presented using fold-change values transformed to log 2 format compared with control (Co). Transcriptional change values along with details of ANOVA and standard error are shown in Supplementary Table 7B. The values for A 4h (Figure 2B) was included as a representative for heat stress. Green and red colors indicate down- and up-regulation of the genes, respectively. “‡” Symbol represents control genes obtained from previous publication (see Supplementary Table 1B for detailed information).

The expression pattern of the candidate Acd genes in response to low temperature, dehydration, salinity, abscisic acid (ABA), hydrogen peroxide, and salicylic acid (SA) treatments were examined by qPCR. We also included expression analysis of 7 more genes of this family for heat, dehydration and salt stress from publicly available microarray data. The combined analysis showed that all the candidate genes exhibit significant differential expression in response to several stress conditions (Figure 4). The maximum percentage of genes showing up-regulation is in response SA treatment 77% (17/22) and least for cold 50% (11/22). Nearly ~76% (22/29) genes are up-regulated in response to heat, 73% (16/22) in response to hydrogen peroxide as well as ABA, and 62% (18/29) genes are more abundant in response to salt stress and dehydration stress (Supplementary Figure 3). Two UAP genes, *SlAcd27.2-CVIII* and *SlAcd23.1-CIX*, exhibit up-regulation in all the above seven treatments. In addition, *SlAcd17.9-CIII* is significantly differentially regulated in all the treatments including six up-regulations and one down-regulation (heat). Moreover, *SlHsp15.6-CI, SlHsp23.8-MTI*, and *SlHsp26.5-PX* are up-regulated in at least six stresses/treatments, including heat stress. This suggests a universal role these proteins play in imparting tolerance to varied stresses. Similarly, in *O. sativa* comparison of sHsp gene expression under heat, drought, salinity, and low temperature revealed highly similar and overlapping response and regulation patterns under different stresses (Hua et al., 2009). Muoki et al. (2012a) also reported up-regulation of sHsp genes in response to drought, salinity, and heat in *Camellia sinensis*. Heat induced sHSPs have also been shown to protect tomato fruit from subsequent chilling injury (Sabehat et al., 1998). However, some sHsp genes also exhibit specific expression patterns in response to distinct stresses (Siddique et al., 2008). Our expression profiling reveals that 7 genes (5 sHsp and 2 UAP) are induced by both low and high temperature. Three genes (2 sHsp and 1 UAP) are up-regulated by heat but down-regulated by low temperature. Conversely, only 1 gene (*SlAcd17.9-CIII*) is up-regulated by low temperature stress but is down-regulated by high temperature. Interestingly, all sHsp genes show up-regulation in heat and dehydration stress except *SlHsp17.3-CII* which is down-regulated upon dehydration and *SlHsp24.5-CI* and *SlHsp21.5-P* that are not affected by dehydration stress. On the other hand, unlike sHsp genes, the UAP group of genes show similar trend in gene expression for salt, ABA, hydrogen peroxide, and SA treatment; the expression being up-regulated in all except *SlAcd15*.7-CI (Figure 4; Supplementary Table 7B). In contrast to tomato, the *O. sativa* Acd genes were not affected significantly by cold, salt, dehydration, and anoxia stress (Sarkar et al., 2009). We find that at least 5 Acd genes (1 sHsp and 4 UAP) are up-regulated in response to low temperature, dehydration and the phytohormone, ABA. The former two stresses and salt stress also commonly up-regulates 5 genes, of which the genes belonging to UAP class are common between the two sets. Numerous studies have also previously shown the existence of cross-talk between low temperature, dehydration and salinity stress signaling processes, with ABA acting as a key molecule connecting the three stress responses (Seki et al., 2002; Fujita et al., 2006).

To gain further insights into the mechanism responsible for the transcriptional regulation of Acd genes, *in-silico* analysis of the putative promoter sequences was performed. A number of stress-related motifs responsive to heat, drought, and low temperature stresses were identified. Several *cis*-regulatory elements involved in response to hormones like ABA, auxin, ethylene, gibberellic acid, methyl jasmonate, and SA were also identified (Supplementary Table 9). There are 136 methyl jasmonate-, 109 TC-rich repeats, 89 heat shock element (HSE), 89 MBS binding sites, 79 ABA-, 55 SA-, 46 gibberellic acid-, 19 ethylene-, 22 auxin-, and 19 low temperature-response elements in the promoters of Acd genes. Overall, the results indicate that expression profiles of Acd genes and the presence of *cis*-elements in the promoters are in good agreement (Figures 2, 4). *SlHsp26.5-PX* has the highest number of *cis*-regulatory elements of 23 including elements responsive to heat, dehydration and ABA (Supplementary Table 9) and expression analysis also exhibited high induction by the respective cues (Figures 2, 4). In contrast, there are exceptions for the presence of the *cis*-regulatory elements and induction of the gene by the respective cues. For example, *SlHsp16.1-CIII* and *SlHsp23.8-MTI* exhibit up-regulation in response to heat stress (Figure 2) even though their promoters lack the canonical HSE (Supplementary Table 9); conversely HSE is found in *SlAcd17.9-CIII*, yet heat induction was not detected. Sung et al. (2001) also reported similar results for the Hsp70 genes in *A. thaliana* and suggested that induction of Hsp70 genes might result from the function of a complex array of *cis*-regulatory elements.

These results reiterate that the Acd genes have both shared as well as distinct regulatory modules in response to various stresses and hormones and suggest possible combinatorial interactions between different stress- and hormone-inducible pathways in regulating the expression of these genes.

### Tomato Acd genes are differentially expressed in various developmental stages

**Figure 5:**
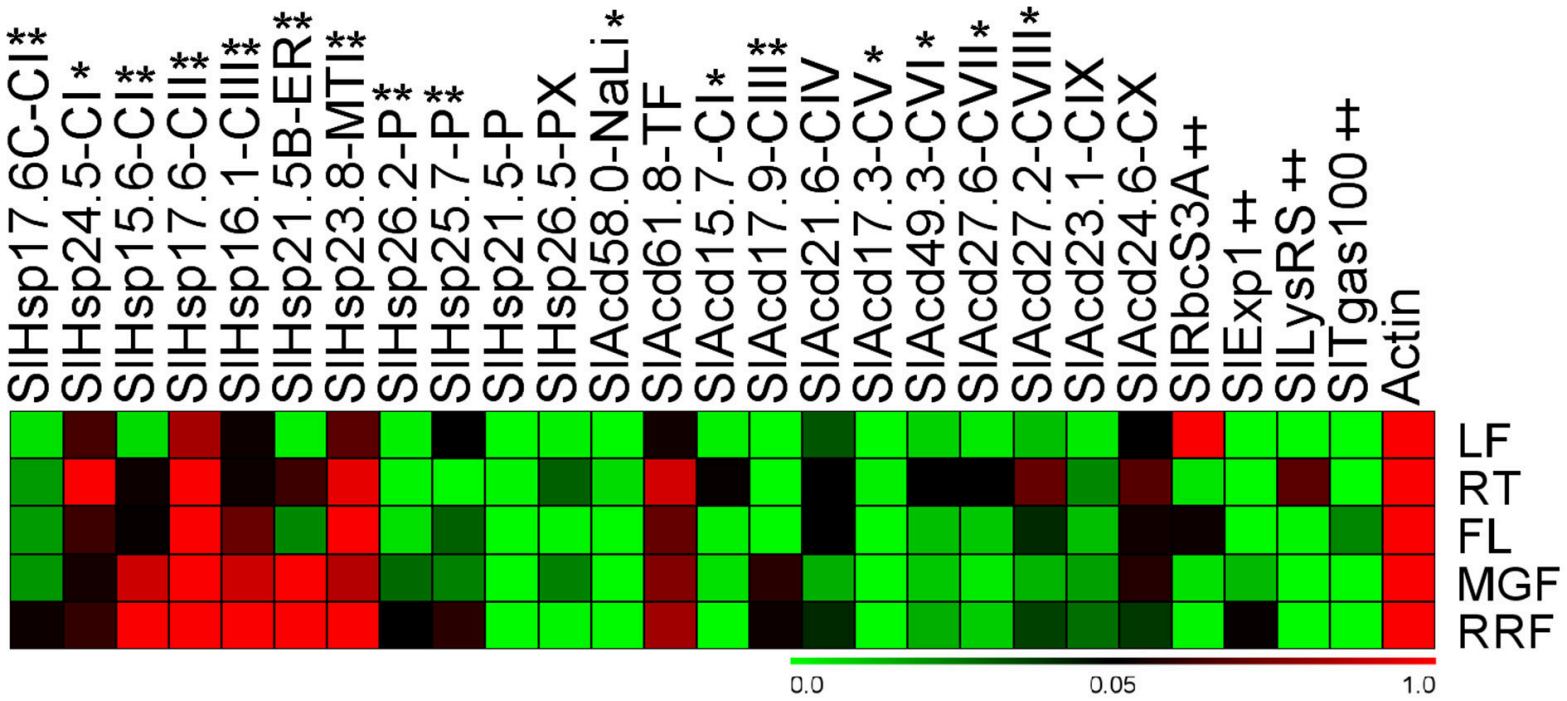
**Heat map representation for the tomato Acd gene expression profiles in various organs**. Expression values were calculated using the 2^−ΔCt^ method, relative to respective *Actin* gene expression. Transcriptional change values along with details of ANOVA and standard error are shown in Supplementary Table 7C. Single asterisk, genes with substantially more expression in roots than other tissues; double asterisks, genes expressed predominantly in fruit tissues in comparison to LF and RT. Genes highly or weakly expressed in the tissues are colored red and green, respectively with reference to *Actin* gene expression which is taken as 1. Leaf (LF), root (RT), flower (FL), mature-green fruit (MGF), red-ripe fruit (RRF). “‡” Symbol represents control genes obtained from previous publication (see Supplementary Table 1B for detailed information).

In addition to being stress inducible, many stress related-genes, including Acd genes exhibit developmental-specific expression (Nover and Scharf, 1997; Neta-Sharir et al., 2005; Sarkar et al., 2009). Expression analysis in various organs of field grown tomato bushes was investigated for Acd genes using qPCR and publicly available microarray expression data for 7 more tomato genes (Figure 5; Supplementary Figure 4). Using qPCR, the relative transcript abundance of the 22 candidate genes was scored against the housekeeping gene *Actin*. *SlHsp15.6-CI, SlHsp16.1-CIII*, and *SlHsp23.8-MTI* accumulates much higher transcripts in red-ripe fruit stage in comparison to *Actin*. Moreover, transcripts are very high in fruits (both mature-green and red-ripe stages) in, *SlHsp21.5B-ER* and *SlHsp17.6-CII* in comparison to *Actin* gene. *SlHsp17.6-CII* is also highly expressed in the root and flower and *SlHsp23.8-MTI* is also highly expressed in flower in comparison to the control gene. The mRNA levels are elevated in root tissue only as compared to *Actin* expression levels for *SlHsp24.5-CI*. The expression of *SlHsp21.5-P, SlAcd58.0-NaLi*, and *SlAcd17.3-CV* is low in all organs (negligible in some cases), the expression of *SlAcd15.7-CI* is negligible in the red-ripe fruit, while *SlAcd17.9-CIII* expression is low in root, leaf, and flower and *SlHsp25.7-P* expression is low in roots, in comparison to *Actin* expression. This suggests that the expression of these genes is responsive to specific cues or developmental stages. The remaining genes exhibited ubiquitous expression in all the organs.

A comparison (fold-change) of transcripts between vegetative (leaf and root) and reproductive (flower and two fruit stages) tissues revealed that all the genes are significantly up-regulated in roots and/or reproductive tissues (Figure 5; Supplementary Table 7C). The genes, *SlHsp24.5-CI* and *SlAcd49.3-CVI*, are root-specific, in addition 6 more genes (5 from qPCR and 1 from microarray data having *p* < 0.05) show highest up-regulation in roots in comparison to other tissues (Figure 5, marked by single asterisk, Supplementary Table 7C). Two genes (*SlHsp16.1-CIII* and *SlHsp26.2-P*) are specific to the three reproductive tissues while seven more genes (Figure 5, marked by double asterisks Supplementary Table 7C) are expressed predominantly in fruit (mature green/red-ripe) in comparison to both the vegetative controls. In addition, the transcripts of *SlHsp17.7A-CI, SlHsp17.3-CII, SlHsp16.1-PX*, and *SlAcd16.7-CX* are also more abundant in fruit as compared to other organs as per microarray data (Supplementary Figure 4). The chip-based data also revealed that *SlAcd57.6-NaLi* expresses primarily in all aerial parts with highest expression in stem and leaf, whereas *SlAcd32.3-CIX* has highest expression in leaf and flower (Supplementary Figure 4).

Expression analysis in various organs of field grown tomato bushes showed that Acd genes exhibited varied expression in different organs. In tomato, HSP21 a chloroplast sHSP (*SlHsp26.2-P* in the present study), has been shown to be induced by heat treatment in leaves, as well as under normal growth conditions in developing fruits during the transition of chloroplasts to chromoplasts, promoting color changes during fruit maturation (Lawrence et al., 1997; Neta-Sharir et al., 2005). We also find that *SlHsp26.2-P* is up-regulated in response to heat (Figure 4), its transcripts are abundantly present in fruit tissues, with highest expression in red-ripe stage (Figure 5). Similarly, *SlHsp25.7-P* exhibits higher expression in red-ripe as compared to mature-green fruit (Figure 5); this gene is earlier characterized as Viscosity 1 (vis1), that exhibited chaperone function and contribute to physiochemical properties of juice, including pectin depolymerization, by reducing thermal denaturation of depolymerizing enzymes during daytime elevated temperatures (Ramakrishna et al., 2003). On the contrary, *SlAcd17.9-CIII* exhibits higher expression in mature-green fruit followed by red-ripe fruit as compared to the expression in root, flower, and leaf (Figure 5); further the gene is down-regulated by heat stress (Figure 4) suggesting a prominent role in early fruit development stages than the later ripening stages. Moreover, *SlAcd17.9-CIII* ortholog is absent in *A. thaliana* and *O. sativa* (Figure 1; Supplementary Table 4) further suggesting a possible role toward fleshy fruit development in tomato.

### *SlHsp24.5-CI* and *SlHsp26.5-PX* play prominent role in thermo-tolerance in the field

**Figure 6:**
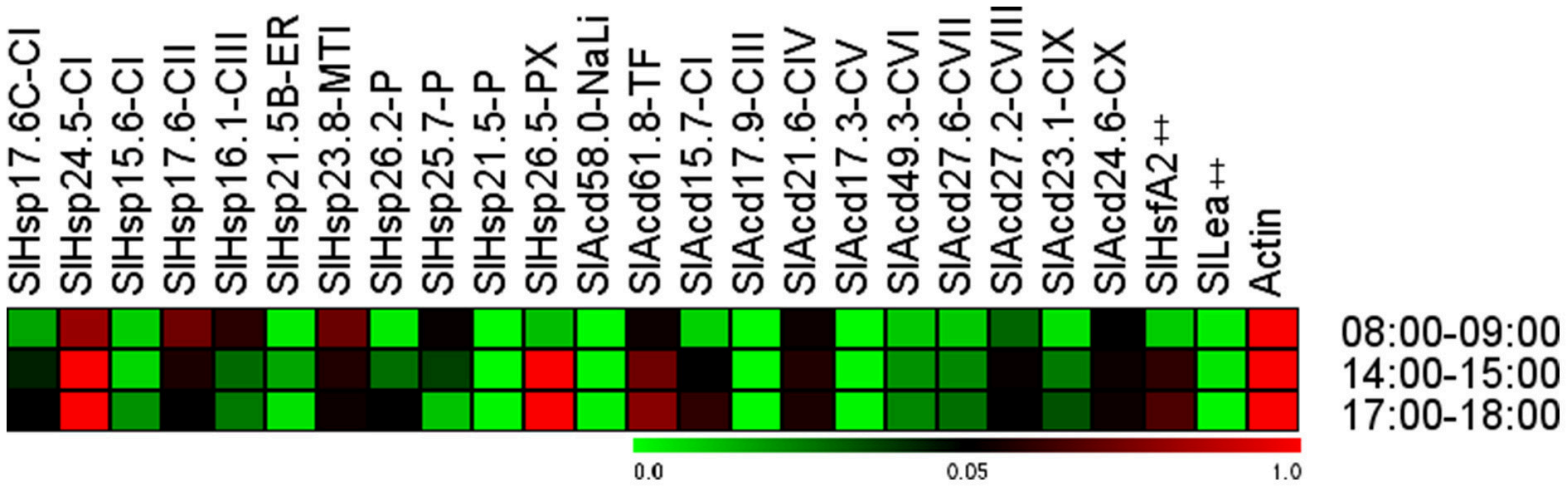
**Heat map representing expression pattern of tomato Acd genes during different daytime in field conditions**. Tomato leaves were collected from field at the indicated times. Expression values were calculated using the 2^−ΔCt^ method, relative to respective *Actin* gene expression. Transcriptional change values along with details of ANOVA and standard error are shown in Supplementary Table 7D. Genes highly or weakly expressed are colored red and green, respectively with reference to *Actin* gene expression which is taken as 1. “‡” Symbol represents control genes obtained from previous publication (see Supplementary Table 1B for detailed information).

Plant sHSPs are produced in response to a wide array of environmental cues (Vierling, 1991; Sun et al., 2002; Hua et al., 2009; Sarkar et al., 2009). We, therefore, decided to evaluate the *in planta* expression pattern of tomato sHsp and other Acd genes in response to diurnal variations of environmental cues such as temperature, light intensity, relative humidity that continuously fluctuate under natural conditions. These parameters were recorded at three different time-points of the day (Supplementary Table 10) and the relative abundance of transcripts was evaluated in comparison to *Actin* gene (Figure 6).

Temperature was highest between 14:00 and 15:00 h (afternoon), followed by 17:00 and 18:00 h (evening) and 08:00 and 09:00 h (morning). Light intensity was highest in afternoon, followed by morning and least in evening. Relative humidity was highest in morning and low in afternoon and evening. Despite variations in the above three parameters during the day, we did not find any significant difference in the relative water content of the plant tissues at the three time-points analyzed (Supplementary Table 10) suggesting that despite these diurnal variations, the plant is not experiencing any osmotic stress. Most of the Acd genes exhibit higher expression at afternoon and evening as compared to morning, which correlates with the increasing field temperatures (Figure 6; Supplementary Table 10), a result similar to that obtained previously (Ramakrishna et al., 2003). Interestingly, the expression of *SlHsp17.6-CII, SlHsp16.1-CIII*, and *SlHsp23.8-MTI* is highest in morning when the temperature is least among the three time-points analyzed. Earlier, Siddique et al. (2003) have also reported similar results, they have shown that *SlHsp16.1-CIII* is heat inducible, however, the synthesis of its protein diminishes at higher temperature due to deficiency in the splicing of its precursor mRNA. We find that *SlHsp21.5-P, SlAcd58.0-NaLi, SlAcd17.9-III*, and *SlAcd17.3-V* are very low expressing as compared to the housekeeping gene. We notice a rapid rise in transcripts for the sHsp genes *SlHsp24.5-CI* and *SlHsp26.5-PX* at afternoon and evening; their transcript abundance even crossing the levels of the housekeeping genes (Figure 6; Supplementary Table 7D) suggesting their prominent role in conferring tolerance to high temperature experienced by the plant. Moreover, we have shown that *SlHsp24.5-CI* and *SlHsp26.5-PX* encode for holdase chaperone (Figure 3). These genes may be excellent candidates for enhancing thermo-tolerance of tomato plants with no or little penalty on its growth and development. Over-expressing *CsHSP17.5* (the most abundant cytosolic sHsp-CI in chestnut stems and seeds) in hybrid poplar has been shown to significantly improve basal thermo-tolerance as well as plant performance and yield including callus growth, bud production, shoots proliferation, plantlets rooting, and survival (Merino et al., 2014). Similarly, accumulation of sHsps in tomato (through altering the master regulator HsfA1 as well as HsfA2) increases heat tolerance in tomato (Mishra et al., 2002).

Our data suggests that the coordinated expression of different ACD proteins may function in synchrony as a chaperone network protecting cellular machinery against thermal denaturation during the daily cycles of daytime rise in temperature experienced by tomato plant in the field.

## Conclusion

The study identifies a compendium of 50 tomato proteins as putative members belonging to the Acd gene family. Expansion of this gene family by way of tandem and segmental duplications appears to be instrumental in defining the functional diversity of its members in tomato. Expression analysis of 22 genes selected from all the 18 classes of this family identified in the study by qPCR showed that most of the sHsp and some UAP genes are highly up-regulated in response to high temperature. Further, the heat stress regime itself influences their expression pattern; while most genes are regulated similarly in both basal and acclimated tissues, some genes are more responsive during acclimation process and others are unique to basal treatment. Some of these genes also appear to be important in the recovery phase, once the heat is removed and may play important role in plant survival. Besides high temperature, the expression of the Acd gene members was also analyzed in response to other abiotic stresses and plant hormones. The expression profiling reveals a co-ordinated inter-play of these genes in response to various stresses and/or phytohormones highlighting a complex network of cross-talk between these genes for plant protection and growth. Several Acd genes were found to be highly expressing in fruit, root, and flower as compared to leaf signifying the role of this gene family in plant development too. Further, three SlHsps exhibited chaperone activity *in-vitro*. In view of the significance of this gene family in response to various stresses and roles in development, this study provides valuable information for selecting promising candidate genes for abiotic stress tolerance and further functional validations *in*-*planta*.

## Author contributions

AP performed all the experiments and compiled the study. SR did the localization and duplication experiments and helped in tissue generation and protein assays. SM was involved in the design, supervision, interpretation, and preparation of the study.

### Conflict of interest statement

The authors declare that the research was conducted in the absence of any commercial or financial relationships that could be construed as a potential conflict of interest.

## Abbreviations

ACD: α-crystallin domain
CDD: conserved domain database
HSE: heat shock element
NaLi: sodium-lithium
qPCR: quantitative polymerase chain reaction
REST: relative expression software tool
RWC: relative water content
SA: salicylic acid
sHSP: small heat shock protein
SGN: Sol Genomics Network
UAP: uncharacterized ACD protein.
