# Supplementary material for: The α-Crystallin Domain Containing Genes: Identification, Phylogeny and Expression Profiling in Abiotic Stress, Phytohormone Response and Development in Tomato (Solanum lycopersicum)
Source: Front Plant Sci. 2016 Mar 31;7:426. doi: 10.3389/fpls.2016.00426 (PMC4814718; doi:10.3389/fpls.2016.00426)
Supplement: Supplementary file 1 [file Table1.PDF]

Supplementary Table 1: Primer sequences and information.

(A) List of primer sequences for SlAcd genes used for qPCR analysis.

Sr. no	Gene	Sequence (5’-3’)
1	SlHsp17.6C-CI F	TGCTAAACACTCCTCCAATGA
2	SlHsp17.6C-CI R	TGCTTGCTTGCTTTCGATT
3	SlHsp24.5-CI F	GCAGGAACCATCACCATATT
4	SlHsp24.5-CI R	GTGGAGTAGTACCACCAAAT
5	SlHsp15.6-CI F	AAGATGTGCAGCTTATTGAGGA
6	SlHsp15.6-CI R	GATCGACGAAGAAGCAGTGA
7	SlHsp17.6-CII F	CTCTGTTTTGATGGTTTGTGGT
8	SlHsp17.6-CII R	CCCATGGCTATTGTAACATTCA
9	SlHsp16.1-CIII F	ATACGAAGCAACGGGAAGAG
10	SlHsp16.1-CIII R	TCGCATTTGGCAGTAATAGC
11	SlHsp21.5B-ER F	GCGGAGAGAGGAAGAAAGAA
12	SlHsp21.5B-ER R	AGGCAAACGAAATTGTCT
13	SlHsp23.8-MTI F	AATTAGGAGCGTGAGCCAAT
14	SlHsp23.8-MTI R	GTGCAGCTACGAATGGAGAA
15	SlHsp26.2-P F	AACTTTCACGATTCCTCCACA
16	SlHsp26.2-P R	AATTGCTTACAGAAGGAGGT
17	SlHsp25.7-P F	GAGAGGTCTAGTAACAAGTCATTCA
18	SlHsp25.7-P R	GAGCAAAGGTGAGTGATGAAAA
19	SlHsp21.5-P F	GTCGCTTGCACCGTTAACTT
20	SlHsp21.5-P R	CTCAAATCGAGCGAAGGAAC
21	SlHsp26.5-PX F	GCCTTTGAAGGACCAATGTT
22	SlHsp26.5-PX R	CGAGGCCTAGCAAATTTAGG
23	SlAcd58.0-NaLi F	TGGAACGTAGCCCTACTCCT
24	SlAcd58.0-NaLi R	TCCAAGTCCAATCTCCATCA
25	SlAcd61.8-TF F	ATTCACAGGCAACCGTATCA
26	SlAcd61.8-TF R	GGAAGAGCAGGACTGGAATC
27	SlAcd15.7-CI F	CCCAAAAACAACCAAACACC
28	SlAcd15.7-CI R	CTTGGAAGAAAGAAGAGGATTTAGG
29	SlAcd17.9-CIII F	AGTTTTTGCGCCTGTGAATA
30	SlAcd17.9-CIII R	GGACACAATTGGCCATTAAA
31	SlAcd21.6-CIV F	CATTTGTGTCTCCGTTGAGG
32	SlAcd21.6-CIV R	CCACTCTCCAGTCCTTCGTC
33	SlAcd17.3-CV F	GGATTGAGTCAGGACGATGA
34	SlAcd17.3-CV R	CCTACCTCGTGCCTGTTCTT
35	SlAcd49.3-CVI F	TCCAAGCACCTGATGACTGT
36	SlAcd49.3-CVI R	TTCATCGGCTAACTGCTTTG
37	SlAcd27.6-CVII F	GGCATTCTTCATGTCAAACAA
38	SlAcd27.6-CVII R	CGTTGATTGTGGCTCGTCT

39	SlAcd27.2-CVIII F	GTTCGTCGTCACTGCTCATT
40	SlAcd27.2-CVIII R	TTGATCACTTCATGCCCAAT
41	SlAcd23.1-CIX F	AGGTTGCCTGCTTCGACTAT
42	SlAcd23.1-CIX R	CGTCACTACAAGCTCACCGT
43	SlAcd24.6-CX F	GCCGGAATCTACCTCACTGT
44	SlAcd24.6-CX R	TCACCATAATTCGGCAAAGA
45	Actin F	GCAAAGGCAGAGTATGACGA
46	Actin R	GCATCTCTGGTCCAGTAGGAA
F: Forward primer; R: Reverse primer.		

(B) Primer sequences and detailed information for the control genes used for qPCR analysis.

Sr. no	Gene	Sequence (5'-3')	Accession no.	Reference
1	SlPr-5F	GCAACAACTGTCCATACACC	NM001247422	Molinari et al. (2014)
2	SlPr-5R	AGACTCCACCACAATCACC		
3	SlCatF	TGATCGCGAGAAGATACCTG	Sl12g094620	Zhou et al. (2014)
4	SlCatR	CTTCCACGTTCATGGACAAC		
5	SlDhnF	AAAGCGCACCAAGTCTCATA	XM004238363.2	Weiss and Egea-Cortines (2009)
6	SlDhnR	CAACAGTCTCTTCAACGGATG		
7	SlHsfA2F	CCTTTAGAGATCACTCTTGTGTTCC	X67601	Giorno et al. (2010)
8	SlHsfA2R	CGACCATAACTCTATCCTTCCC		
9	SlTas14F	GTATATTGGCGGCCATGC	X51904	Godoy et al (1990)
10	SlTas14R	CGGGACACCATAACACAC		
11	SlLeaF	CTGATGCTGTGAAGCACACTT	SGN-U213276	Gong et al. (2010)
12	SlLeaR	TGAACACTTGAATCCATCAAGA		
13	SlApxF	TTTTCATCCTGGGAGACAGG	Solyc09g007270	Unpublished data
14	SlApxR	GTGTCCAAAAACCTCCCTCA		
15	SlUPF0603F	GGTCCATTCGGAAATGACTG	Solyc01g098640	Unpublished data
16	SlUPF0603R	AGCGATGCAACAGGAAAGTT		
17	SlRbsS3AF	AATGCCAATTTTTAATTATGTAA	NM001309210	Wanner and Gruissem (1991)
18	SlRbsS3AR	ACAAGTTTCAGAAGCAGATT		
19	SlLysRSF	CAGAAGAGAGCTATGTGATGCCTA	X94451	Giritch et al. (1997)
20	SlLysRSR	GTCGTCTCCTAATTGTCTGTCCTT		
21	SlTgas100F	GTTGTGCATCCAAATGCAAG	AJ133599	Heuvel et al. (2002)
22	SlTgas100R	CCACCTTATACATATCATGGCCCTAC		
23	SlExp1F	GGAAAAGAAATCGAGTCCTCAC	U82123	Rose et al. (1997)
24	SlExp1R	CCACTTCAACCTTTCTCTCCAT		
F: Forward primer; R: Reverse primer.				

References for control genes

Giorno, F., Wolters-Arts, M., Grillo, S., Scharf, K.-D., Vriezen, W.H., and Mariani, C. (2010) Developmental and heat stress-regulated expression of HsfA2 and small heat shock proteins in tomato anthers. *J. Exp. Bot.* 61, 453-462.

Giritch, A., Herbik, A., Balzer, H-J., Ganal, M., Stephan, U.W., and Bäumlein, H. (1997) Root-specific iron-regulated gene of tomato encodes a lysyl-tRNA-synthetase-like protein. *Eur. J. Biochem.* 244, 310-317.

Godoy, J.A., Pardo, J.M., and Pintor-Toro, J.A. (1990) A tomato cDNA inducible by salt stress and abscisic acid: nucleotide sequence and expression pattern. *Plant Mol. Biol.* 15, 695-705.

Gong, P., Junhong, Z., Li, H., Yang, C., Zhang, C., Zhang, X., et al. (2010) Transcriptional profiles of drought-responsive genes in modulating transcription signal transduction, and biochemical pathways in tomato. *J. Exp. Bot.* 61, 3563-3575.

Heuvel, K.J.V., Lipzig, R.H.V., Barendse G.W., and Wullems, G.J. (2002) Regulation of expression of two novel flower-specific genes from tomato (*Solanum lycopersicum*) by gibberellin. *J. Exp. Bot.* 53, 51-59.

Molinari, S., Fanelli, E., and Leonetti, P. (2014) Expression of tomato salicylic acid (SA)-responsive pathogenesis-related genes in Mi-1-mediated and SA-induced resistance to root-knot nematodes. *Mol. Plant Pathol.* 15, 255-264.

Rose, J.K.C., Lee, H.H., and Bennett, A.B. (1997) Expression of a divergent expansin gene is fruit-specific and ripening-regulated. *Proc. Natl Acad. Sci. USA* 94, 5955-5960.

Wanner, L. A., and Gruissem, W. (1991) Expression dynamics of the tomato rbcS gene family during development. *Plant Cell* 3, 1289-1303.

Weiss, J., and Egea-Cortines, M. (2009) Transcriptomic analysis of cold response in tomato fruits identifies dehydrin as a marker of cold stress. *J. Appl. Genet*. 50, 311-319.

Zhou, J., Wang, J., Li, X., Xia, X.J., Zhou, Y.H., Shi, K., et al. (2014) H_2O_2 mediates the crosstalk of brassinosteroid and abscisic acid in tomato responses to heat and oxidative stresses. *J. Exp. Bot.* 65, 4371-4383.

(C) Gene specific primers used for full-length cloning and protein expression

Gene	Outer primers designed from un-translated region of the cDNA	Nested primer sequences to clone the ORF
SlHsp24.5-CI	F: CAAGACATCAACTCAACTCCACAT<br>R: GTGGAGTAGTACCACCAAAT	F: CCATATGTCTCTCATGCCTGTGTTTGG<br>R: CCTCGAGTTAATTAATTTGAACAGATC
SlAcd15.7-CI	F: CCCAAAAACAACCAAACACC<br>R: ATCTTGAAATCCCTCTCCCTAAAC	F:CCATATGGAATTCTCAACTTTCCATC<br>R: CCTCGAGTCAAGCAGCACTAGCACCAAG
	Primer sequences to clone the ORF	
SlHsp17.6-CI	F: CCATATGTCTCTGATCCCAAGAATTTTC<br>R: CCTCGAGTTAACCAGAAATCTCAATGGAC	
SlHsp26.5-PXII	F: GGAATTCCATATGGAGAGTCAAATTGTTCGACG<br>R: CCGCTCGAGTCAAAGTTTCGGGATTGTAATC	

F: Forward primer; R: Reverse primer. The *Nde*I (CATATG) and *Xho*I (CTCGAG) sites are underlined
