# Supplementary material for: The α-Crystallin Domain Containing Genes: Identification, Phylogeny and Expression Profiling in Abiotic Stress, Phytohormone Response and Development in Tomato (Solanum lycopersicum)
Source: Front Plant Sci. 2016 Mar 31;7:426. doi: 10.3389/fpls.2016.00426 (PMC4814718; doi:10.3389/fpls.2016.00426)
Supplement: Supplementary file 3 [file Table3.doc]

| Class | Gene name | Locus ID | Gene Ontology (GO) terms at SGN |
| --- | --- | --- | --- |
| UAP I | *SlAcd15.7-CI* | Solyc02g080410.2 | none |
| UAP III | *SlAcd17.9-CIII* | Solyc04g072250.2 | GO:0042803-protein homodimerization activity |
| UAP IV | *SlAcd21.6-CIV* | Solyc07g064020.2 | none |
| UAP V | *SlAcd17.3-CV* | Solyc03g113170.1 | none |
|  | *SlAcd23.8-CV* | Solyc03g113180.2 | none |
| UAP VI | *SlAcd49.3-CVI* | Solyc01g096960.2 | none |
|  | *SlAcd39.4-CVI* | Solyc01g096980.1 | none |
| UAP VII | *SlAcd25.7-CVII* | Solyc01g009200.2 | none |
|  | *SlAcd23.8-CVII* | Solyc01g009220.2 | none |
|  | *SlAcd26.8-CVII* | Solyc09g007140.2 | none |
|  | *SlAcd15.5-CVII* | Solyc10g076880.1 | none |
|  | *SlAcd27.6-CVII* | Solyc11g071560.1 | none |
| UAP VIII | *SlAcd37.0-CVIII* | Solyc04g071490.2 | none |
|  | *SlAcd27.2-CVIII* | Solyc12g056560.1 | none |
| UAP IX | *SlAcd32.3-CIX* | Solyc03g005190.2 | GO:0051536-iron-sulfur cluster binding  GO:0022900-electron transport chain |
|  | *SlAcd23.1-CIX* | Solyc06g054150.1 | GO:0005515-protein binding |
|  | *SlAcd16.0-CIX* | Solyc06g084220.1 | GO:0005515- protein binding |
|  | *SlAcd18.0-CIX* | Solyc09g065370.1 | GO:0005515-protein binding |
|  | *SlAcd11.3-CIX* | Solyc09g082150.1 | none |
| UAP X | *SlAcd54.0-CX* | Solyc01g098790.1 | none |
|  | *SlAcd24.6-CX* | Solyc01g098810.2 | none |
|  | *SlAcd16.7-CX* | Solyc04g082720.2 | none |
|  | *SlAcd21.6-CX* | Solyc04g082740.2 | none |

Supplementary Table 3: Gene Ontology search for 23 uncharacterized ACD proteins (UAP).
