# Supplementary material for: The α-Crystallin Domain Containing Genes: Identification, Phylogeny and Expression Profiling in Abiotic Stress, Phytohormone Response and Development in Tomato (Solanum lycopersicum)
Source: Front Plant Sci. 2016 Mar 31;7:426. doi: 10.3389/fpls.2016.00426 (PMC4814718; doi:10.3389/fpls.2016.00426)
Supplement: Supplementary file 4 [file Table4.PDF]

Supplementary Table 4: Comparative list of Acd genes of tomato, *A. thaliana* and *O. sativa*.

Class	Tomato		*A. thaliana*†		*O. sativa*†	
	Gene name	SGN Locus	Gene name	TAIR Locus	Gene name	RGAP Locus
CI sHSP	*SlHsp17.7A-CI*<br>*SlHsp17.6A-CI*<br>*SlHsp17.6B-CI*<br>*SlHsp17.6C-CI*<br>*SlHsp9.1-CI*<br>*SlHsp24.5-CI*<br>*SlHsp15.2-CI*<br>*SlHsp17.7B-CI*<br>*SlHsp27.1-CI*<br>*SlHsp15.6-CI*	Solyc06g076520.1<br>Solyc06g076540.1<br>Solyc06g076570.1<br>Solyc06g076560.1<br>Solyc07g045610.1<br>Solyc09g011710.2<br>Solyc09g015000.2<br>Solyc09g015020.1<br>Solyc10g086680.1<br>Solyc02g093600.2	*AtHsp17.8-CI*<br>*AtHsp17.6A-CI*<br>*AtHsp17.6C-CI*<br>*AtHsp17.6B-CI*<br>*AtHsp17.4-CI*<br>*AtHsp18.1-CI*<br>*AtHsp18.5-CI*	AT1G07400<br>AT1G59860<br>AT1G53540<br>AT2G29500<br>AT3G46230<br>AT5G59720<br>AT2G19310	*OsHsp16.9C-CI*<br>*OsHsp16.9A-CI*<br>*OsHsp16.9B-CI*<br>*OsHsp17.9A-CI*<br>*OsHsp17.4-CI*<br>*OsHsp18.0-CI*<br>*OsHsp17.7-CI*<br>*OsHsp17.8-CI*<br>*OsHsp16.6-CI*<br>*OsHsp17.9B-CI*	OS01g04360<br>OS01g04370<br>OS01g04380<br>OS03g15960<br>OS03g16020<br>OS03g16030<br>OS03g16040<br>OS02g48140<br>OS01g04340<br>OS01g04350
CII sHSP	*SlHsp17.3-CII*<br>*SlHsp17.6-CII*	Solyc08g062340.2<br>Solyc08g062450.1	*AtHsp17.6-CII*<br>*AtHsp17.7-CII*	AT5G12020<br>AT5G12030	*OsHsp18.0-CII*<br>*OsHsp19.0-CII*	OS01g08860<br>OS02g12610
CIII sHSP	*SlHsp16.1-CIII*	Solyc03g123540.2	*AtHsp17.4-CIII*	AT1G54050	*OsHsp18.6-CIII*	OS02g54140
ER sHSP	*SlHsp21.6-ER*<br>*SlHsp21.5A-ER*<br>*SlHsp21.5B-ER*	Solyc01g102960.2<br>Solyc03g113930.1<br>Solyc11g020330.1	*AtHsp22.0-ER*	AT4G10250	*OsHsp23.2-ER*<br>*OsHsp21.8-ER*	OS04g36750<br>OS11g13980
MT sHSP	*SlHsp23.8-MTI*	Solyc08g078700.2	*AtHsp23.6-MTI*<br>*AtHsp23.5-MTI*<br>*AtHsp26.5-MTII*	AT4G25200<br>AT5G51440<br>AT1G52560	*OsHsp24.0-MTI*<br>*OsHsp26.2-MTI*<br>*OsHsp23.6-MTII*	OS02g52150<br>OS06g11610<br>OS02g10710
P sHSP	*SlHsp26.2-P*<br>*SlHsp25.7-P*<br>*SlHsp21.5-P*	Solyc03g082420.2<br>Solyc05g014280.2<br>Solyc08g078710.1	*AtHsp25.3-P*	AT4G27670	*OsHsp26.7-P*<br>*OsHsp27.6-P*	OS03g14180<br>OS05g23140
P -like sHSP					*OsHsp21.5-P-like*<br>*OsHsp38.4-P-like*<br>*OsHsp21.0-P-like*	OS09g17660<br>OS10g07200<br>OS10g07210

PX sHSP	*SlHsp16.1-PX*<br>*SlHsp26.5-PX*	Solyc04g014480.2<br>Solyc07g055720.2	*AtHsp15.7-PX*<br>*AtHsp31.2-PX*	AT5G37670<br>AT1G06460	*OsHsp16.0-PX*	OS06g14240
NaLi	*SlAcd57.6-NaLi*<br>*SlAcd57.1-NaLi*<br>*SlAcd58.0-NaLi*	Solyc04g011460.1<br>Solyc10g086420.1<br>Solyc11g066090.1	*AtAcd55.8-NaLi*<br>*AtAcd55.3-NaLi*<br>*AtAcd56.6-NaLi*	AT2G37570<br>AT3G12570<br>AT5G02480	*OsAcd59.7-NaLi*<br>*OsAcd58.3-NaLi*<br>*OsAcd59.0-NaLi*	OS01g05790<br>OS01g62300<br>OS03g16910
TF	*SlAcd61.8-TF*<br>*SlAcd48.0-TF*	Solyc04g082820.2<br>Solyc12g094730.1	*AtAcd44.3-TF*<br>*AtAcd48.0-TF*<br>*AtAcd86.6-TF*	AT1G20910<br>AT1G76510<br>AT2G17410	*OsAcd52.9-TF*<br>*OsAcd50.4-TF*	OS02g48370<br>OS06g41730
UAP I	*SlAcd15.7-CI*	Solyc02g080410.2	*AtAcd15.4-CI*	AT4G21870	*OsAcd18.8-CI*	OS07g33350
UAP II					*OsAcd18.8-CII*	OS02g03570
UAP III	*SlAcd17.9-CIII*	Solyc04g072250.2				
UAP IV	*SlAcd21.6-CIV*	Solyc07g064020.2	*AtAcd21.7-CIV*	AT5G54660	*OsAcd22.2-CIV*	OS05g42120
UAP V	*SlAcd17.3-CV*<br>*SlAcd23.8-CV*	Solyc03g113170.1<br>Solyc03g113180.2			*OsAcd18.1-CV*<br>*OsAcd29.4-CV*<br>*OsAcd34.9-CV*	OS01g40530<br>OS01g40550<br>OS05g51440
UAP VI	*SlAcd49.3-CVI*<br>*SlAcd39.4-CVI*	Solyc01g096960.2<br>Solyc01g096980.1	*AtAcd54.2-CVI*<br>*AtAcd41.3-CVI*	AT3G10680<br>AT5G04890		
UAP VII	*SlAcd25.7-CVII*<br>*SlAcd23.8-CVII*<br>*SlAcd26.8-CVII*<br>*SlAcd15.5-CVII*<br>*SlAcd27.6-CVII*	Solyc01g009200.2<br>Solyc01g009220.2<br>Solyc09g007140.2<br>Solyc10g076880.1<br>Solyc11g071560.1	*AtAcd20.4-CVII*<br>*AtAcd25.1-CVII*<br>*AtAcd28.1-CVII*	AT1G54400<br>AT2G27140<br>AT5G20970	*OsAcd22.6-CVII*<br>*OsAcd41.4-CVII*<br>*OsAcd31.8-CVII*<br>*OsAcd18.0-CVII*<br>*OsAcd19.4-CVII*	OS03g06170<br>OS03g45330<br>OS03g45340<br>OS10g30162<br>OS10g30180
UAP VIII	*SlAcd37.0-CVIII*<br>*SlAcd27.2-CVIII*	Solyc04g071490.2<br>Solyc12g056560.1	*AtAcd28.7-CVIII*	AT1G76770		
UAP IX	*SlAcd32.3-CIX*<br>*SlAcd23.1-CIX*<br>*SlAcd16.0-CIX*<br>*SlAcd18.0-CIX*<br>*SlAcd11.3-CIX*	Solyc03g005190.2<br>Solyc06g054150.1<br>Solyc06g084220.1<br>Solyc09g065370.1<br>Solyc09g082150.1	*AtAcd22.1-CIX*<br>*AtAcd16.9-CIX*	AT3G22530<br>AT4G14830	*OsAcd16.9-CIX*<br>*OsAcd17.4-CIX*<br>*OsAcd18.3-CIX*	OS03g06390<br>OS07g07670<br>OS10g29549

UAP X	*SlAcd54.0-CX*<br>*SlAcd24.6-CX*<br>*SlAcd16.7-CX*<br>*SlAcd21.6-CX*	Solyc01g098790.1<br>Solyc01g098810.2<br>Solyc04g082720.2<br>Solyc04g082740.2	*AtAcd51.9-CX*<br>*AtAcd39.4-CX*<br>*AtAcd22.3-CX*<br>*AtAcd15.5-CX*	AT1G20870<br>AT1G54840<br>AT1G54850<br>AT1G76440		
UAP XI			*AtAcd13.0-CXI*<br>*AtAcd27.7-CXI*<br>*AtAcd57.5-CXI*<br>*AtAcd81.4-CXI*<br>*AtAcd25.4-CXI*<br>*AtAcd14.7-CXI*<br>*AtAcd28.9-CXI*	AT1G12180<br>AT2G03020<br>AT4G16560<br>AT4G16550<br>AT4G16540<br>AT5G47600<br>AT5G47590		

† *A. thaliana* ACD containing genes were reported previously in Siddique et al. (2008) and Bondino et al. (2012), while *O. sativa* sequences were reported in Sarkar et al. (2009) and Bondino et al. (2012).
