# Supplementary material for: The α-Crystallin Domain Containing Genes: Identification, Phylogeny and Expression Profiling in Abiotic Stress, Phytohormone Response and Development in Tomato (Solanum lycopersicum)
Source: Front Plant Sci. 2016 Mar 31;7:426. doi: 10.3389/fpls.2016.00426 (PMC4814718; doi:10.3389/fpls.2016.00426)
Supplement: Supplementary file 8 [file Table8.PDF]

Supplementary Table 8: Prediction of putative interacting proteins partner for SlAcd15.7-CI (Solyc02g080410.2.1) using STRING (http://string-db.org/; software for predicting protein-protein interactions).

Input Protein: SlAcd15.7-CI (Solyc02g080410.2.1)		
Predicted Functional Partners		
Protein identity	Annotation	Score*
Solyc07g043560.2.1	heat shock 70 kDa protein 17-like (890 aa)	0.777
Solyc12g042060.1.1	ATP-dependent Clp protease ATP-binding subunit clpA homolog CD4B, chloroplastic (923 aa)	0.749
Solyc11g006650.1.1	uncharacterized LOC101244337 (844 aa)	0.749
Solyc10g082000.1.1	uncharacterized LOC101267467 (724 aa)	0.749
Solyc09g055230.2.1	uncharacterized LOC101247060 (1075 aa)	0.749
Solyc07g018070.2.1	uncharacterized LOC101267323 (1022 aa)	0.749
Solyc07g006540.2.1	uncharacterized LOC101262825 (1052 aa)	0.749
Solyc06g082560.1.1	uncharacterized protein (854 aa)	0.749
Solyc06g051460.2.1	uncharacterized LOC101252191 (1009 aa)	0.749
Solyc06g011400.2.1	chaperone protein ClpB4, mitochondrial-like (529 aa)	0.749
Solyc06g011380.2.1	uncharacterized protein (205 aa)	0.749
Solyc06g011370.2.1	uncharacterized protein (298 aa)	0.749
Solyc05g055200.2.1	uncharacterized LOC101263518 (723 aa)	0.749
Solyc03g118360.2.1	uncharacterized protein (162 aa)	0.749
Solyc03g118340.2.1	ATP-dependent Clp protease ATP-binding subunit clpA homolog CD4A, chloroplastic (926 aa)	0.749
Solyc03g117950.2.1	chaperone protein ClpD, chloroplastic-like (964 aa)	0.749
Solyc03g115230.2.1	chaperone protein ClpB1-like (911 aa)	0.749
Solyc02g088610.2.1	heat shock protein (980 aa)	0.749
Solyc01g007260.2.1	uncharacterized LOC101261145 (846 aa)	0.749
*Score of ≥0.7 represents high confidence		
