# Supplementary material for: The α-Crystallin Domain Containing Genes: Identification, Phylogeny and Expression Profiling in Abiotic Stress, Phytohormone Response and Development in Tomato (Solanum lycopersicum)
Source: Front Plant Sci. 2016 Mar 31;7:426. doi: 10.3389/fpls.2016.00426 (PMC4814718; doi:10.3389/fpls.2016.00426)
Supplement: Supplementary file 10 [file Table10.PDF]

Supplementary Table 10: Values of the air temperatures, light intensity, relative humidity and relative water content at the time of tissue harvest during different daytime in field conditions (for Figure 6).

Time	Temperature ± SE (°C)	Light Intensity ± SE (Lux)	Relative humidity ± SE (%)	Relative water content ± SE (RWC, %)
8:00-9:00	33.17±1.50[†]	57700.00±3346.24[†]	37.63±2.47[†]	85.90±5.18[*]
14:00-15:00	40.23±0.99[†]	79087.50±4752.67[†]	18.50±0.89[†]	82.30±3.77[*]
17:00-18:00	38.53±0.89[†]	14687.50±2518.43[†]	20.13±1.20[†]	88.77±3.66[*]

[†]Standard error (SE); with n=16, reading were recorded at interval of every 4 minutes for 1 h for 2 consecutive days.

[*]Standard error (SE); with n=3, samples were harvested at interval of every 20 minutes for 1 h for two consecutive days
