# Supplementary material for: The α-Crystallin Domain Containing Genes: Identification, Phylogeny and Expression Profiling in Abiotic Stress, Phytohormone Response and Development in Tomato (Solanum lycopersicum)
Source: Front Plant Sci. 2016 Mar 31;7:426. doi: 10.3389/fpls.2016.00426 (PMC4814718; doi:10.3389/fpls.2016.00426)

Supplementary Figure 1: SDS-PAGE of the eluted recombinant protein. (A) SlHsp17.6C-CI; (B) SlHsp24.5-CI; (C) SlHsp26.5-PX; (D) SlAcd15.7-CI. kDa: kilodalton, M: protein ladder, E: eluate. Arrows indicates the purified fraction used for holdase chaperone assay.

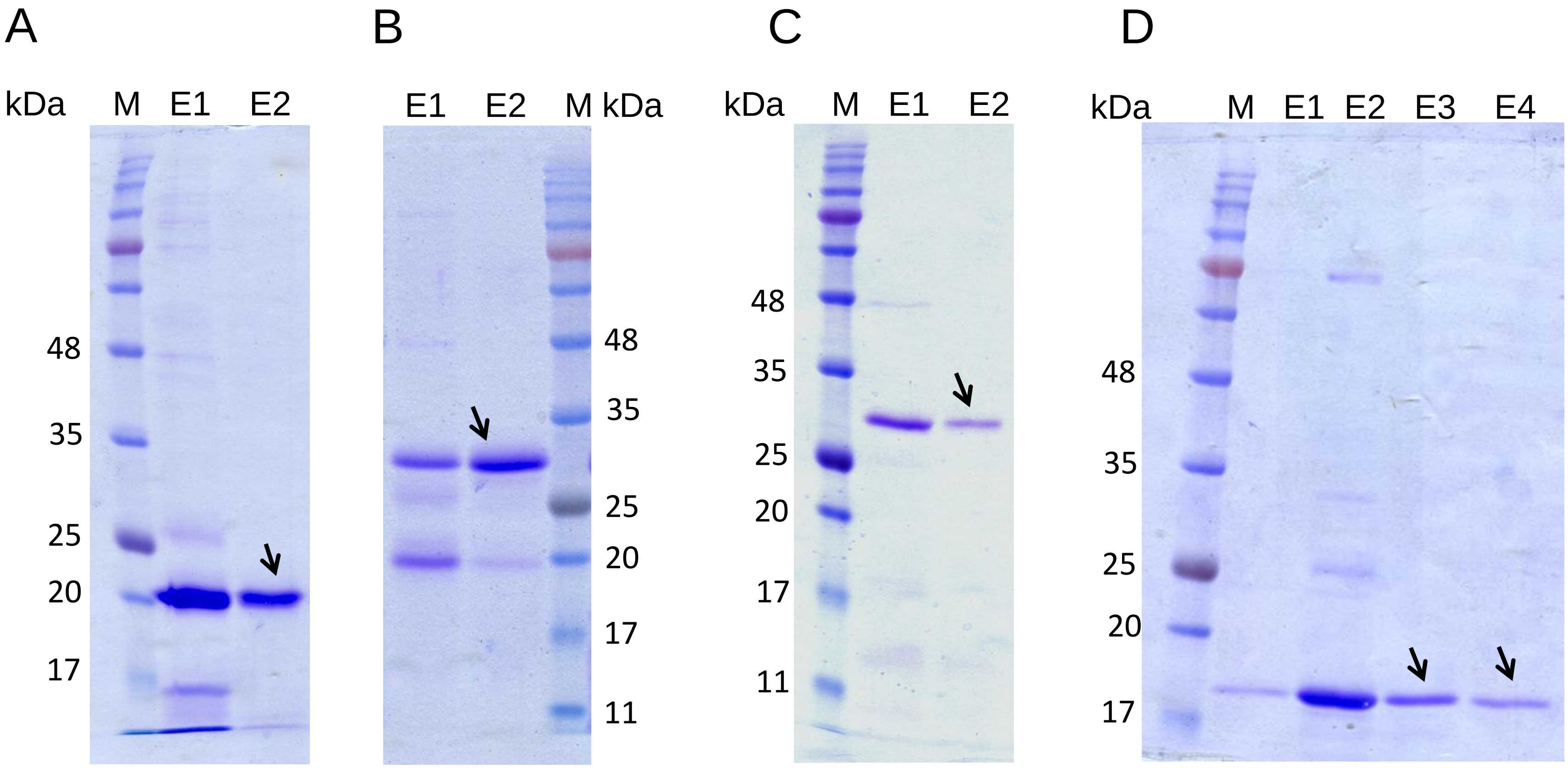

Supplement: Supplementary file 11 [file Presentation1.PDF]
