# Supplementary material for: The α-Crystallin Domain Containing Genes: Identification, Phylogeny and Expression Profiling in Abiotic Stress, Phytohormone Response and Development in Tomato (Solanum lycopersicum)
Source: Front Plant Sci. 2016 Mar 31;7:426. doi: 10.3389/fpls.2016.00426 (PMC4814718; doi:10.3389/fpls.2016.00426)
Supplement: Supplementary file 12 [file Presentation2.PDF]

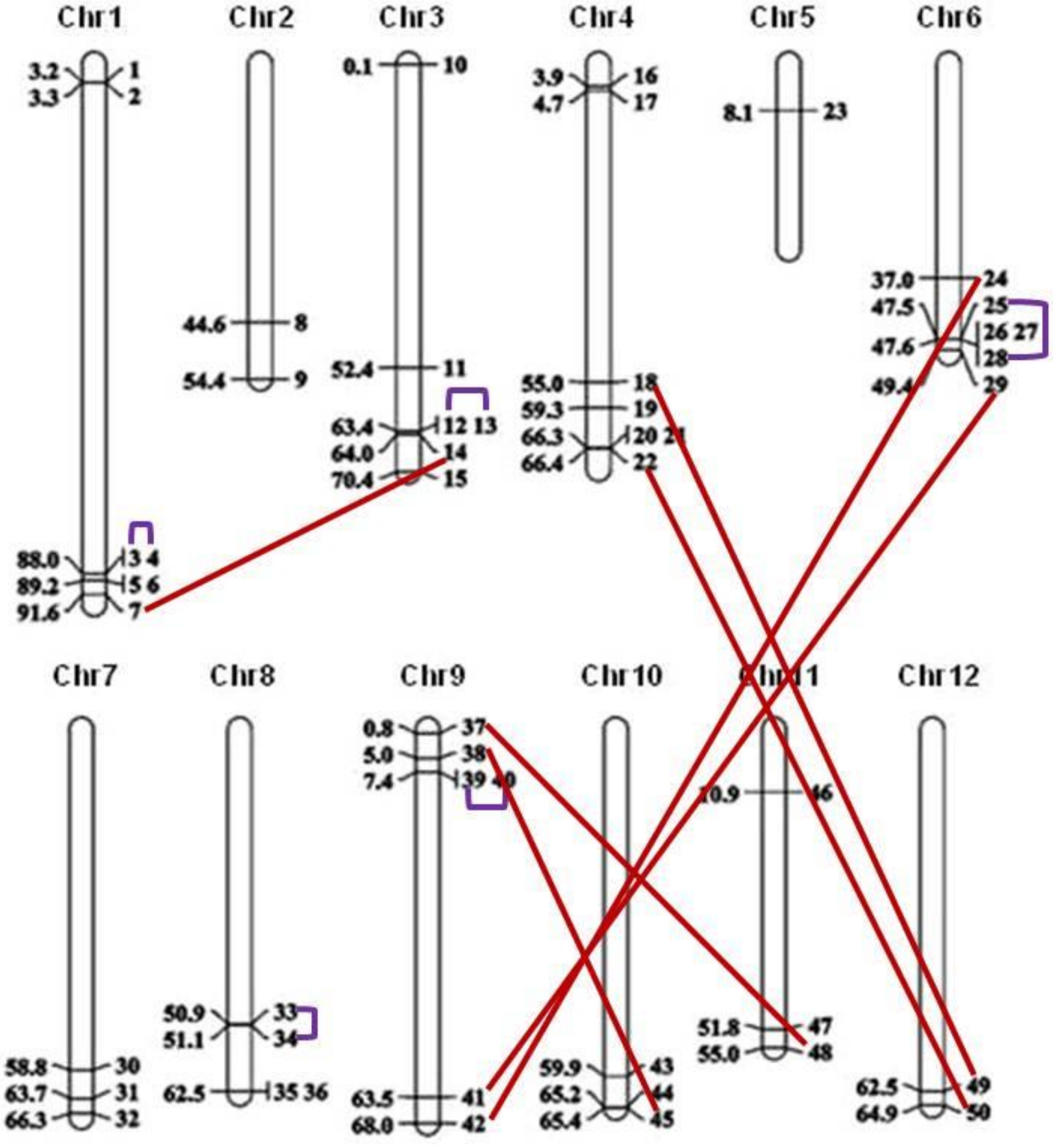


Supplementary Figure 2: Chromosomal locations and duplication events of tomato Acd genes. Acd genes (numbered 1 to 50) are indicated on the right side of each chromosome and their approximate position on the chromosome in Mb on the left. The gene names and their corresponding designated number IDs are provided in Supplementary Table 5. The segmentally duplicated genes are connected by solid lines in red, and the tandem duplicated gene clusters are highlighted by purple brackets.
