# Supplementary material for: The α-Crystallin Domain Containing Genes: Identification, Phylogeny and Expression Profiling in Abiotic Stress, Phytohormone Response and Development in Tomato (Solanum lycopersicum)
Source: Front Plant Sci. 2016 Mar 31;7:426. doi: 10.3389/fpls.2016.00426 (PMC4814718; doi:10.3389/fpls.2016.00426)
Supplement: Supplementary file 13 [file Presentation3.PDF]

Treatments	HT 1(0)	LT 3(0)	DH 1(1)	NaCl 1(1)	H_2O_2 2(0)	ABA 1(0)	SA 0(0)
HT 17(5)		0	0	0	0	0	0
LT 11(0)	7		1	0	0	0	0
DH 13(5)	15	8		0	0	0	0
NaCl 17(1)	13	8	9		1	0	0
H_2O_2 16(0)	11	8	9	15		1	0
ABA 16(0)	11	7	8	13	13		0
SA 17(0)	12	8	9	15	15	15	

Supplementary Figure 3: The number of genes regulated by HT, DH, LT, salt, ABA, H_2O_2 and SA. The numbers below the respective cues represent the differentially expressed genes obtained through qPCR, additional genes obtained from microarray are enclosed by brackets. Green and red colors indicate down- and up-regulation of the genes, respectively.
