# Supplementary material for: The α-Crystallin Domain Containing Genes: Identification, Phylogeny and Expression Profiling in Abiotic Stress, Phytohormone Response and Development in Tomato (Solanum lycopersicum)
Source: Front Plant Sci. 2016 Mar 31;7:426. doi: 10.3389/fpls.2016.00426 (PMC4814718; doi:10.3389/fpls.2016.00426)

Supplementary Figure 4: Microarray based expression analysis of 7 tomato Acd genes in different organs and tissues. Single asterisk: gene with substantially more expression in roots than other tissues.

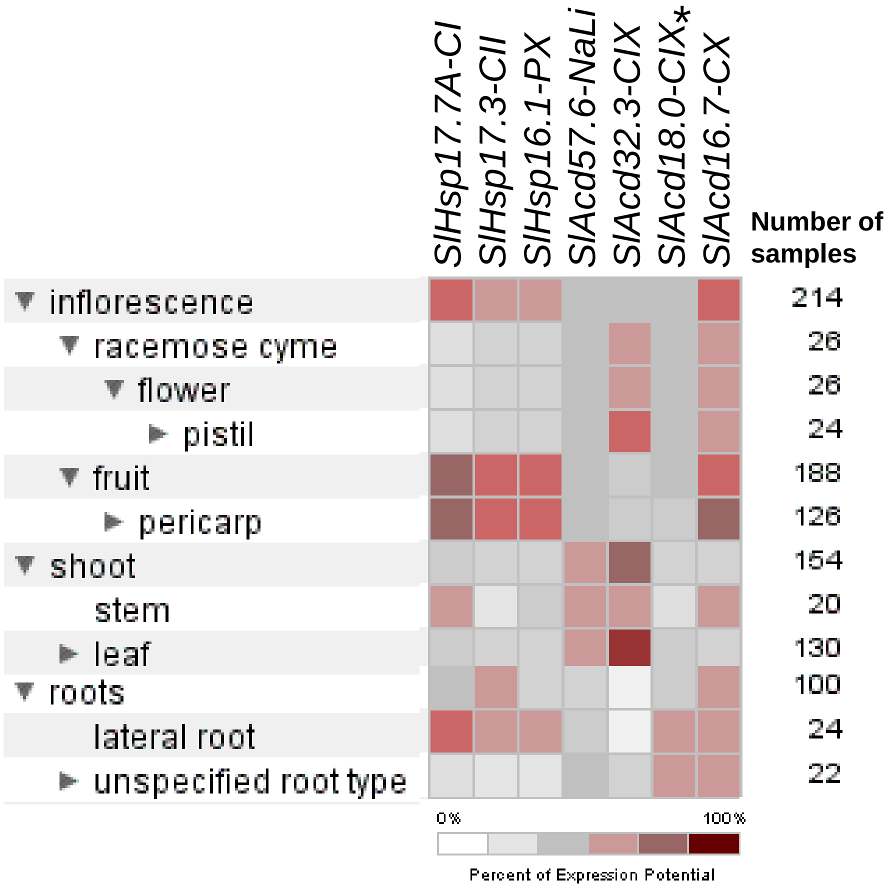

Supplement: Supplementary file 14 [file Presentation4.PDF]
